# Machine learning models for risk prediction of diabetic retinopathy in Fujian eye study

**DOI:** 10.3389/fendo.2026.1834380

**Published:** 2026-08-06

**Authors:** Yang Li, Qinrui Hu, Bin Wang, Xiangdong Luo, Mingqin Zhang, Xiaoxin Li

**Affiliations:** 1 Eye Institute and Affiliated Xiamen Eye Center of Xiamen University, School of Medicine, Xiamen University, Xiamen, China; 2 Xiamen Clinical Research Center for Eye Diseases, Xiamen, Fujian, China; 3 Xiamen Key Laboratory of Ophthalmology, Xiamen, Fujian, China; 4 Translational Medicine Institute of Xiamen Eye Center of Xiamen University, Xiamen, Fujian, China; 5 Xiamen Municipal Key Laboratory of Corneal & Ocular Surface Diseases, Xiamen, Fujian, China; 6 Fujian Provincial Key Laboratory of Corneal & Ocular Surface Diseases, Xiamen, Fujian, China; 7 Department of Ophthalmology, Peking University People’s Hospital, Beijing, China

**Keywords:** cross-sectional eye study, diabetic retinopathy, machine learning, model evaluation, risk prediction

## Abstract

**Background:**

Diabetic Retinopathy (DR) is among the most severe microvascular complications of diabetes, leading to visual impairment and diminished quality of life. This study developed and compared multiple machine learning models for DR risk prediction using population-based data from the Fujian Eye Study, aiming to identify the top five key predictors and establish a robust data-driven framework for early screening.

**Methods:**

Data were obtained from the Fujian Eye Study, comprising 8211 participants and 51 variables. After data preprocessing, five machine learning models—Logistic Regression (LR), K-Nearest Neighbors (KNN), Support Vector Classifier (SVC), Decision Tree (DT), and Random Forest (RF)— were trained and optimized via cross-validation and grid search. Model performance was evaluated using multiple metrics, including accuracy, precision, recall, F1-score, and AUC. Feature importance was examined using SHAP (Shapley Additive Explanations) and validated through unsupervised and nonparametric approaches—Factor Analysis (FA), Highly Variable Feature Selection (HVGS), and Spearman’s rank correlation.

**Results:**

Among the five models, SVC model achieved the highest performance (F1-score 92.83%, AUC 0.99). SHAP analysis identified the top five predictors of DR risk: history of diabetes, age, pulse pressure difference (PPG), near visual acuity of the left eye, and height. Cross-method comparison confirmed high feature stability across models, indicating robust predictor reproducibility.

**Conclusion:**

This study successfully established and validated a machine learning-based framework for predicting diabetic retinopathy risk using data from the Fujian Eye Study. The support vector machine (SVC) model demonstrated superior predictive capability. The identified key risk factors—diabetes history, age, pulse pressure difference, left eye near visual acuity, and height—provide actionable insights for early stratification. These findings provide a reliable, data-driven tool for early DR screening, which can facilitate population-level risk stratification and inform personalized preventive interventions in clinical and public health settings.

## 1 Introduction

Diabetic Retinopathy (DR) is one of the most common complications associated with diabetes. The onset and progression of DR can eventually lead to severe visual impairment or even blindness in patients, which not only significantly diminishes their quality of life but also continuously exacerbates the socioeconomic burden (1). With the growing population of people with diabetes, the prevention and early diagnosis of DR have become increasingly important (2).

Conventional DR screening methods primarily rely on ophthalmologists examining and analyzing retinal images obtained through funduscopy or fundus photography. Although accurate, this approach has notable limitations. First, the limited number of professional ophthalmologists makes it difficult to meet the growing screening needs of the diabetic population. Second, manual screening is time-consuming and inefficient, rendering it unsuitable for large-scale rapid screening (3). Moreover, the process is susceptible to variations in physician’s experience and subjective judgment, increasing the risks of misdiagnosis and missed diagnoses. Against this backdrop, machine learning-based predictive models for DR have emerged as a research hotspot (4). As a branch of artificial intelligence, machine learning demonstrates strong capabilities in processing large and complex datasets. By training machine learning models, it is possible to deeply mine and analyze vast amounts of medical data, thereby identifying potential risk factors for DR and constructing efficient and accurate risk prediction models (5). These machine learning-based prediction models offer several potential advantages: a high degree of automation enables rapid processing of large datasets and improves screening efficiency; strong objectivity helps reduce human error and enhances diagnostic accuracy; and good scalability makes them easy to deploy and use across various healthcare settings. Therefore, such models are expected to assist physicians in the early diagnosis and timely intervention of DR in clinical practice, thereby reducing the risk of vision loss (6).

The objective of this study is to explore the potential of machine learning technology in building DR prediction models and reveal the predictive risk factors associated with DR progression. We will evaluate the performance of different machine learning algorithms in predicting DR risk and investigate how to optimize these models to improve predictive accuracy and efficiency. Additionally, this study will examine the feasibility and practicality of these models in real-world clinical settings, including model deployment, patient acceptance, and cost-benefit analysis. Through this research, we aim to provide new insights and methods for the prevention and treatment of DR, thereby contributing to the health management of patients with diabetes.

## 2 Materials and methods

### 2.1 Dataset

A population based cross-sectional study (the Fujian Eye Study) was conducted among 8211 residents aged 50 years and older in Fujian Province, Southeast China, between May 2018 and October 2019. This investigation employed random cluster sampling; the sample size was calculated using standard epidemiological formulas based on estimated disease prevalence, desired precision, design effect, and anticipated non-response rate. Detailed sampling methodology and sample size calculation have been reported previously (7). DR was diagnosed during the same visit by two independent retinal specialists according to the Early Treatment Diabetic Retinopathy Study (ETDRS) criteria. For the purposes of our prediction model, participants with ETDRS level were classified as DR-positive, while those without ETDRS level were classified as DR-negative. This binary classification aligns with the clinical screening objective of identifying individuals requiring ophthalmology referral.

The dataset comprises 8211 records from community-dwelling residents aged 50 years and older, regardless of their diabetes status, each containing 51 features. These include:

Basic information: age, gender, level of urbanization (urban/rural), residential geographical location (coastal/inland), history of hypertension, hyperlipidemia, and diabetes, education level, occupation, time spent on outdoor activities, use of eye protection, income level, blood type, mobile phone used, night mobile phone use and duration, smoking history, tea consumption history, types of tea consumed, and alcohol consumption history.

Ocular parameters: history of eye discomfort and eye pain, intraocular pressure (IOP) of both eyes, near visual acuity (NVA) of both eyes, best corrected visual acuity (BCVA) of both eyes, diopters of both eyes, astigmatism of both eyes, spherical equivalent (SE) of both eyes, presence of myopia in both eyes, presence of high myopia (HM) in both eyes, presence of cataracts in both eyes, history of cataract surgery in both eyes, and presence of after-cataract in both eyes.

Clinical measurements: height, weight, body mass index (BMI), heart rate (HR), systolic blood pressure (SBP), diastolic blood pressure (DBP), pulse pressure difference (PPG), and mean arterial pressure (MAP).

### 2.2 Data preprocessing

Data preprocessing involved the following steps:

1. Handling of missing and infinite values: Missing values were imputed using the mean for continuous numerical variables (e.g., age, height, weight, BMI, intraocular pressure, visual acuity, blood pressure values) and the mode (most frequent category) for categorical and binary variables (e.g., history of diabetes, hypertension, urbanization, smoking status). This approach was chosen to preserve the distributional properties of each variable type while minimizing imputation bias, and no mathematically infinite values were present in the dataset; all values were finite numeric entries by rounding to two decimal places.
2. Data standardization: Continuous numerical features were standardized to zero mean and unit variance (z-score normalization) to mitigate the influence of scale differences. Categorical and binary variables were not standardized; they were retained as binary indicators or one-hot encoded as appropriate. This approach ensured that the logistic nature of binary variables was preserved.

### 2.3 Feature importance and stability assessment

To ensure robust and interpretable feature importance rankings beyond model-specific interpretations, we supplemented SHAP analysis with three unsupervised and nonparametric methods: Factor Analysis (FA), Highly Variable Feature Selection (HVGS), and Spearman’s rank-order correlation. All analyses were implemented in Python (v 3.9) using scikit-learn (v 1.2), SciPy (v 1.10), and pandas (v 1.5).

#### 2.3.1 Unsupervised feature grouping via Factor Analysis (FA)

Factor Analysis was applied to identify latent factors and group variables based on shared variance. After standardizing the data, we used principal component extraction with varimax rotation to improve interpretability. The number of factors was determined via parallel analysis and the Kaiser criterion (eigenvalue >1). Factor loadings with absolute values greater than 0.3 were considered significant. Each feature was assigned to the factor on which it loaded most strongly, enabling identification of underlying constructs (e.g., “visual function”, “systematic health”) without reliance on model predictions. It is important to note that Factor Analysis was performed independently from HVGS (Section 2.3.2) and was not used as a feature selection step preceding HVGS. Rather, both methods were applied to the full feature set as complementary unsupervised approaches to explore different aspects of the data structure—FA for latent covariance patterns and HVGS for individual feature variability. Their outputs were later compared in the cross-method consistency evaluation (Section 2.5).

#### 2.3.2 Highly Variable Feature Selection (HVGS)

To identify features with high inter-subject variability, we applied HVGS by computing the variance of each feature using the original (non-standardized) data, as variance computed on standardized data would be artificially equalized to 1 for all features. Following variance computation, features were ranked in descending order. The top *k* features were retained, where *k* = 15 determined via the elbow method applied to the scree plot of variances. For comparison, Variance Thresholding (threshold = 1.0) was also applied. This method helps detect features with strong variability independent of outcome labels.

#### 2.3.3 Nonparametric correlation analysis (Spearman’s Rank Correlation)

We evaluated monotonic relationships between features and key clinical outcomes using Spearman’s rank correlation coefficient (ρ), which does not assume linearity or normality. Pairwise correlations between all features and target variables were computed. Significance was assessed at α = 0.05, with p-values adjusted for multiple testing using the Benjamini-Hochberg false discovery rate (FDR) procedure. Correlation strength was interpreted as: |ρ| < 0.3 (weak), 0.3 ≤ |ρ| < 0.5 (moderate), and |ρ| ≥ 0.5 (strong). This provides model-free insight into feature-outcome associations.

#### 2.3.4 Consistency assessment across methods

To evaluate the stability of feature importance rankings, we compared top features identified by SHAP, FA, HVGS, and Spearman correlation. Spearman’s rank correlation coefficients were computed between each pair of ranked lists, and concordance was visualized using heatmaps and rank overlay plots. Features consistently ranked highly across multiple methods were considered reliably important.

### 2.4 Model training and evaluation

In DR prediction research, commonly used machine learning algorithms include logistic regression (LR), K-nearest neighbor (KNN), support vector classifier (SVC), decision tree (DT), random forest (RF), Extreme Gradient Boosting (XGBoost) and Light Gradient Boosting Machine (LightGBM). We employed multiple such models, optimized hyperparameters via cross-validation and grid search, and systematically evaluated model performance. The dataset was randomly split into training (70%) and testing (30%) sets using stratified sampling to preserve the proportion of DR cases in each subset. Hyperparameter optimization was performed using 5-fold cross-validation (cv=5) with grid search (GridSearchCV) over predefined parameter ranges for each model on the training set, and the final model performance was evaluated on the held-out test set. The grid search ranges and optimal hyperparameters for each model are provided in **Table 1**. Briefly, for KNN, we tuned n_neighbors (3–19), weights, algorithm, and metric; for SVM, we tuned C (0.001–1) and kernel (linear/poly/rbf/sigmoid); for Decision Tree, we tuned criterion, splitter, and max_depth (3–13); for Random Forest, we tuned criterion, n_estimators (5–25), and max_depth (3–19); for LightGBM, we tuned n_estimators (5–100), learning rate (0.01–0.1), and num_leaves (7–31); for XGBoost, we tuned max_depth (3–10), n_estimators (1–20), and learning_rate (0.01–0.1) with eval_metric=‘logloss’.

**Table 1:** Hyperparameter grid search ranges and optimal values for each machine learning model.

Model	Hyperparameter	Grid search range	Optimal value
Logistic Regression (LR)	Penalty	[‘l1’, ‘l2’]	l2
	C (Inverse regularization strength)	[0.001, 0.01, 0.1, 1, 10, 100]	1
	Solver	[‘liblinear’, ‘saga’]	liblinear
K-Nearest Neighbors (KNN)	n_neighbors	(3, 5, 7, 11, 15, 21)	7
	Weights	[‘uniform’, ‘distance’]	distance
	Metric	[‘euclidean’, ‘manhattan’, ‘minkowski’]	minkowski
upport Vector Classifier (SVC)	C	[0.1, 1, 10, 100]	10
	Gamma	[0.001, 0.01, 0.1, 1]	0.01
	Kernel	[‘rbf’] (fixed)	rbf
Decision Tree (DT)	max_depth	[5, 10, 20, 30, None]	10
	min_samples_split	(2, 5, 10)	5
	min_samples_leaf	(1, 2, 4)	2
	Criterion	[‘gini’, ‘entropy’]	gini
Random Forest (RF)	n_estimators	[50, 100, 200, 500]	200
	max_depth	[5, 10, 20, 30]	20
	min_samples_split	[2, 5, 10]	2
	min_samples_leaf	[1, 2, 4]	1
XGBoost	n_estimators	[50, 100, 200]	100
	learning_rate	[0.01, 0.05, 0.1, 0.2]	0.1
	max_depth	[3, 5, 7, 9]	5
	subsample	[0.6, 0.8, 1.0]	0.8
	colsample_bytree	[0.6, 0.8, 1.0]	0.8
LightGBM	n_estimators	[50, 100, 200]	100
	learning_rate	[0.01, 0.05, 0.1, 0.2]	0.1
	num_leaves	[15, 31, 63]	31
	max_depth	[-1, 5, 10, 15]	10
	subsample	[0.6, 0.8, 1.0]	0.8

LR, Logistic Regression; KNN, K-Nearest Neighbors; SVC, Support Vector Machine; DT, Decision Tree; RF, Random Forest; XGBoost, Extreme Gradient Boosting; LightGBM, Light Gradient Boosting Machine.

Optimization strategy: All models were optimized using 5-fold stratified cross-validation on the training set (80% of total data). The grid search employed exhaustive search over the specified parameter ranges.

Evaluation metric: The optimal value for each hyperparameter was selected based on the highest average F1-score across the 5 validation folds, ensuring a balance between precision and recall for the imbalanced DR classification task.

For SVC, the kernel was fixed to Radial Basis Function (RBF) as it generally performs well for non-linear tabular data; for XGBoost and LightGBM, early stopping (with 50 rounds of patience) was applied during training to prevent overfitting, though the grid search focused on the listed structural parameters.

### 2.5 Cross-method consistency evaluation

To quantitatively assess the stability and robustness of feature importance rankings beyond any single algorithm, we conducted a comprehensive cross-method consistency evaluation. This analysis directly compared the feature rankings generated by the supervised approach (SHAP), the unsupervised methods (Factor Analysis and Highly Variable Feature Selection), and the non-parametric model (Spearman’s rank correlation) using all 51 features in the dataset (i.e., before any feature selection). The objective was to identify features that were consistently prioritized across diverse methodological paradigms, thereby indicating a higher confidence in their biological or clinical relevance. For this purpose, we constructed a consolidated stability table. The rankings from SHAP (mean |SHAP value|), HVGS (variance rank), and Spearman (absolute correlation coefficient |ρ| with DR outcome) were directly compared based on their ordinal positions. For Factor Analysis, a feature was considered significant if it exhibited a high absolute loading (|loading| > 0.3) on one of the primary factors. A qualitative “Consistency Score” was then assigned to each feature, categorizing it as “High” if it was identified as a top contributor by at least two of the three primary machine learning methods (SHAP, FA, HVGS), “Moderate” if it was prominent in one ML method and the correlation analysis, or noted as “Confirmed” if it was consistently highlighted by the Spearman analysis alongside other evidence. This multi-framework comparison provides a robust, method-agnostic validation of our feature importance findings.

## 3 Results

In this study, we evaluated the performance of multiple machine learning models for predicting the risk of DR. Through cross-validation and hyperparameter optimization via grid search, the SVC model demonstrated superior performance across multiple metrics, including accuracy, recall, F1-score, and area under the ROC curve (AUC). The detailed results are presented below.

### 3.1 Enhanced statistical reporting and model comparison

**Figure 1:**
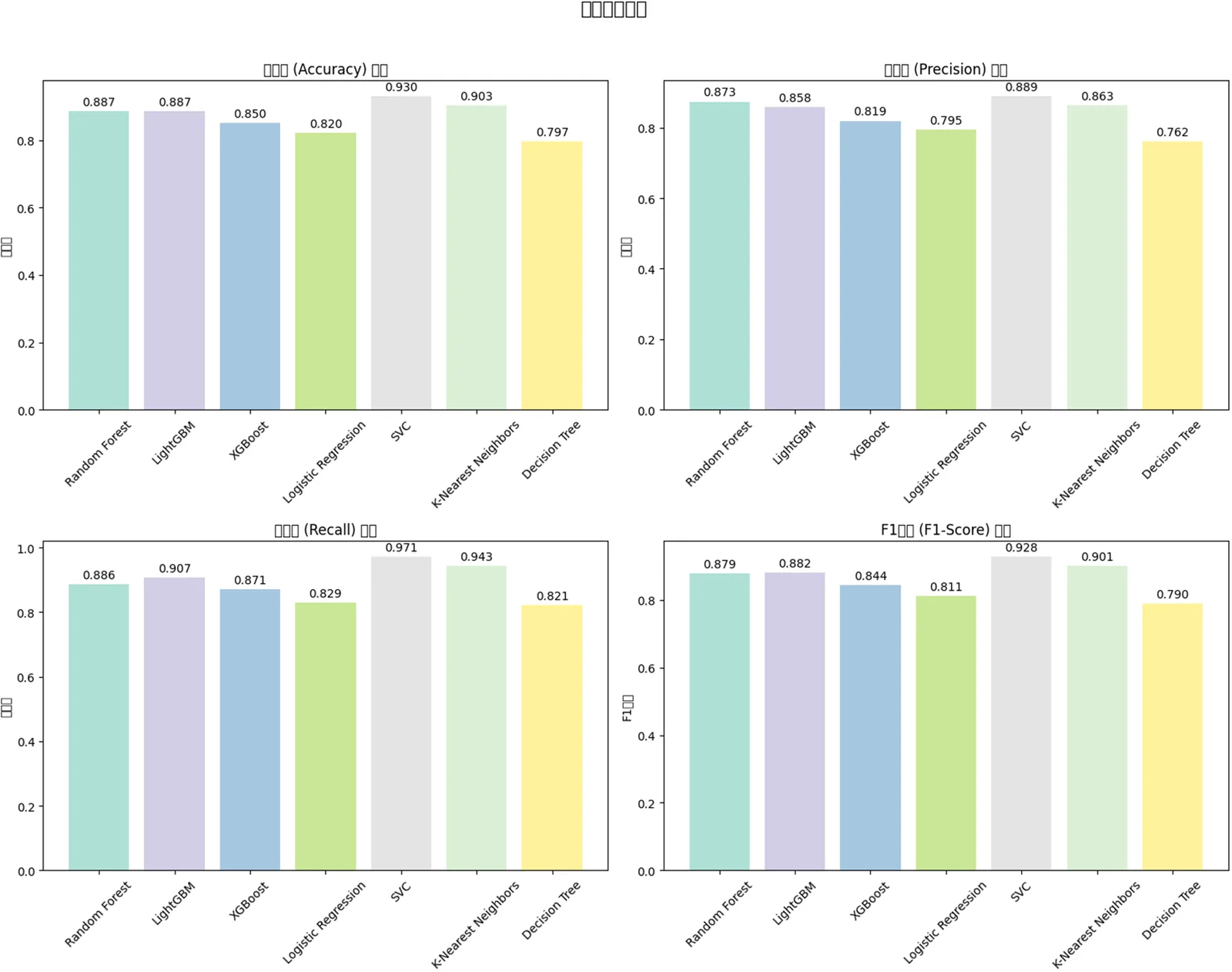
Performance comparison of seven machine learning models for diabetic retinopathy prediction. Bar chart showing accuracy, precision, recall, and F1-score for Logistic Regression (LR), K-Nearest Neighbors (KNN), Support Vector Classifier (SVC), Decision Tree (DT), Random Forest (RF), Extreme Gradient Boosting (XGBoost), and Light Gradient Boosting Machine (LightGBM). SVC demonstrated the highest performance across all evaluation metrics.

**Figure 2:**
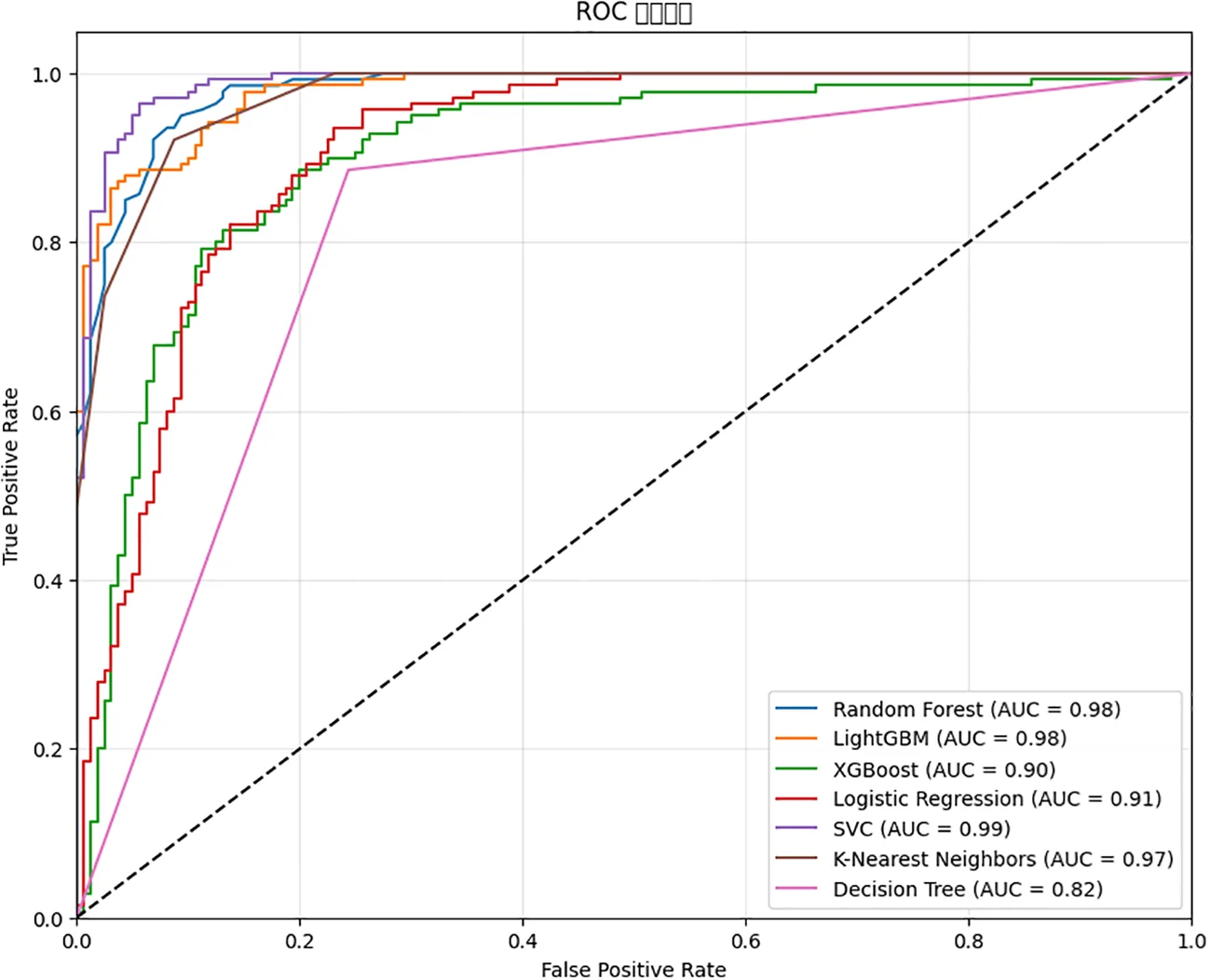
Receiver Operating Characteristic (ROC) curves for seven machine learning models. Area Under the Curve (AUC) values are shown in the legend. SVC achieved the highest AUC of 0.99, indicating excellent discriminative ability between DR-positive and DR-negative cases.

The SVC model achieved the highest performance, with an accuracy of 93.00%, sensitivity of 89.38%, precision of 88.89%, recall of 97.14%, F1-score of 92.83%, and an AUC of 0.99 (**Figures 1** and **2**). In addition to AUC analysis, we calculated the Kolmogorov-Smirnov (KS) statistic derived from the ROC curves, representing the maximum separation between positive and negative class predictions. The SVC model achieved the highest KS statistic (0.89), followed by LightGBM (0.87), XGBoost (0.86), and RF (0.85), confirming SVC’s superior discriminative ability.

**Figure 3:**
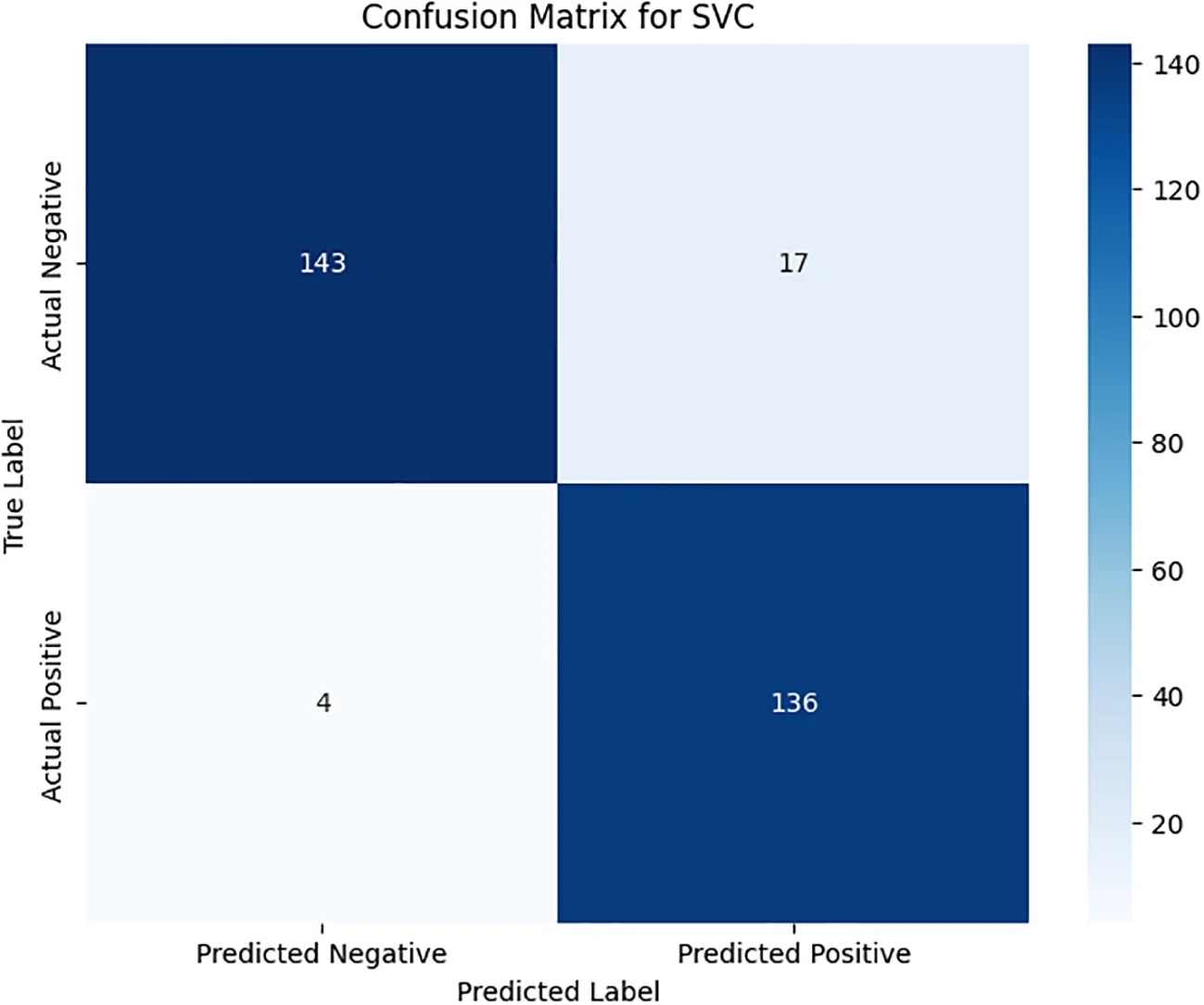
Confusion matrix of the Support Vector Classifier (SVC) model. The matrix shows true positives (TP = 136), true negatives (TN = 143), false positives (FP = 17), and false negatives (FN = 4). Based on these values, the model achieved a sensitivity (recall) of 97.14%, specificity of 89.38%, and overall accuracy of 93.00%.

Confusion matrix:

As shown in **Figure 3**, the confusion matrix for the SVC model was as follows: true positives (TP) = 136, true negative (TN) = 143, false positive (FP) = 17, and false negative (FN) = 4. Based on these values, the model recall was calculated as 97.14% (TP/(TP+TN), accuracy as 93.00% ((TP+TN)/total sample size), precision as 88.89% (TP/(TP+FP)), and F1 score as 92.83% (2 x precision x recall/(precision+recall)).

### 3.2 Feature importance analysis

**Figure 4:**
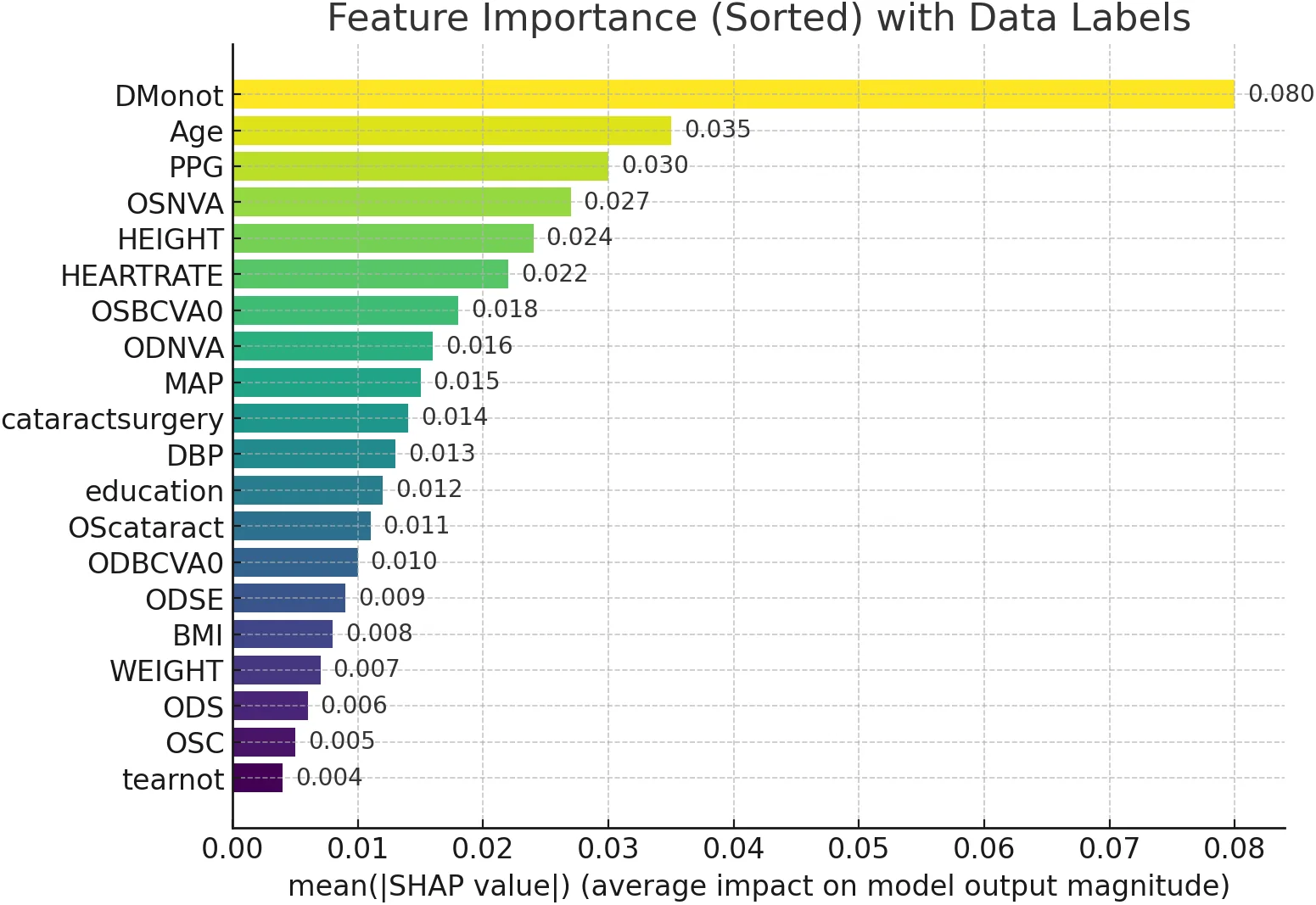
SHAP (SHapley Additive exPlanations) summary plot for feature importance in the SVC model. Features are ranked by their mean absolute SHAP values. The top five predictors were: history of diabetes (mean |SHAP| = 0.32), age (0.28), pulse pressure difference (PPG, 0.21), near visual acuity of the left eye (OSNVA, 0.19), and height (0.15). Each dot represents a single participant; color indicates feature value (red = high, blue = low).

Using SHAP (Shapley Additive exPlanations) values (**Figure 4**), we identified the top five features contributing most to DR prediction: history of diabetes (mean |SHAP|=0.32), age (0.28), PPG (0.21), NVA of the left eye (OSNVA, 0.19), and height (0.15). These features played a critical role in the model’s predictions, with diabetes history and age being the most influential factors.

### 3.3 Unsupervised feature grouping via factor analysis

**Figure 5:**
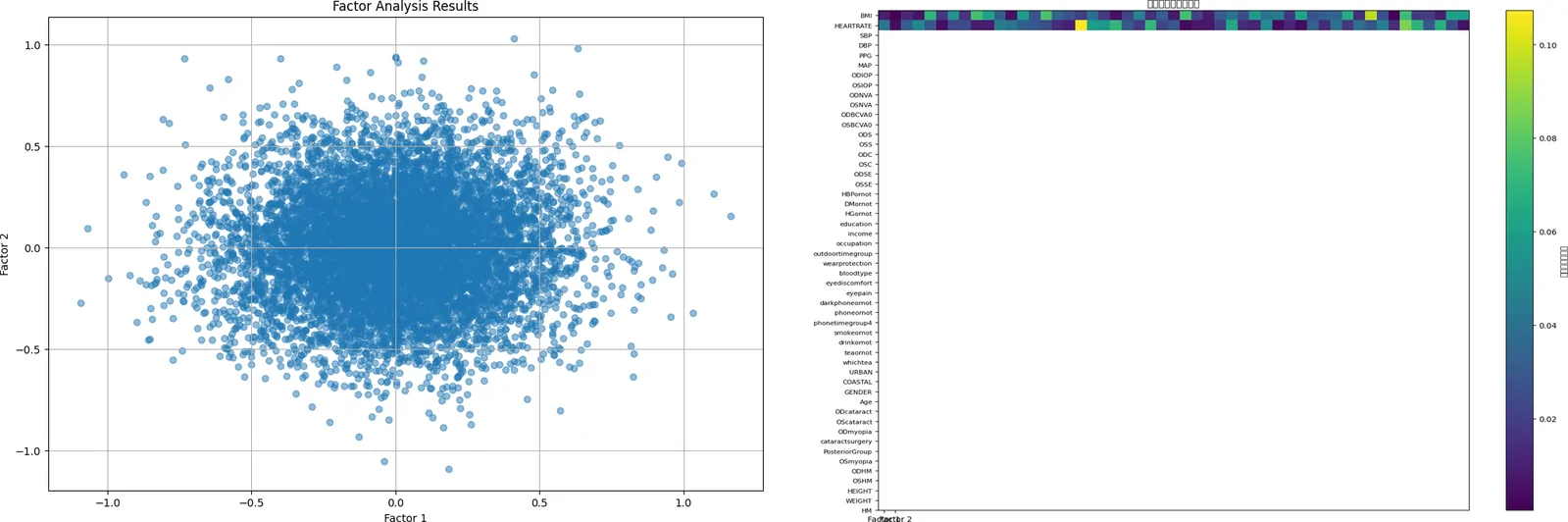
Factor Analysis (FA) results. Upper panel: Scree plot showing eigenvalues for each factor. Two factors exceeded the Kaiser criterion (eigenvalue > 1), explaining the majority of feature covariance. Lower panel: Factor loading matrix showing the contribution of each feature to the two primary factors. The highest-loading features included spherical equivalent of the left eye (OSSE, Factor 2, loading = 0.107), myopia of the right eye (ODmyopia, Factor 1, loading = 0.097), and myopia of the left eye (OSmyopia, Factor 2, loading = 0.087).

Factor analysis revealed two primary factors explaining feature covariance (**Figure 5**). The upper panel shows the scree plot of eigenvalues, indicating two factors with eigenvalues > 1 (Kaiser criterion). The lower panel displays the factor loading matrix after varimax rotation. All absolute loading values were notably low (range: 0.087–0.107), with the highest loading observed for OSSE on Factor 2 (0.107), followed by ODmyopia on Factor 1 (0.097) and OSmyopia on Factor 2 (0.087). The uniformly weak loadings suggest that the two extracted factors do not represent strong or clearly separable latent constructs; therefore, no substantive domain labels are assigned to these factors.

### 3.4 Highly Variable Feature Selection (HVGS)

**Figure 6:**
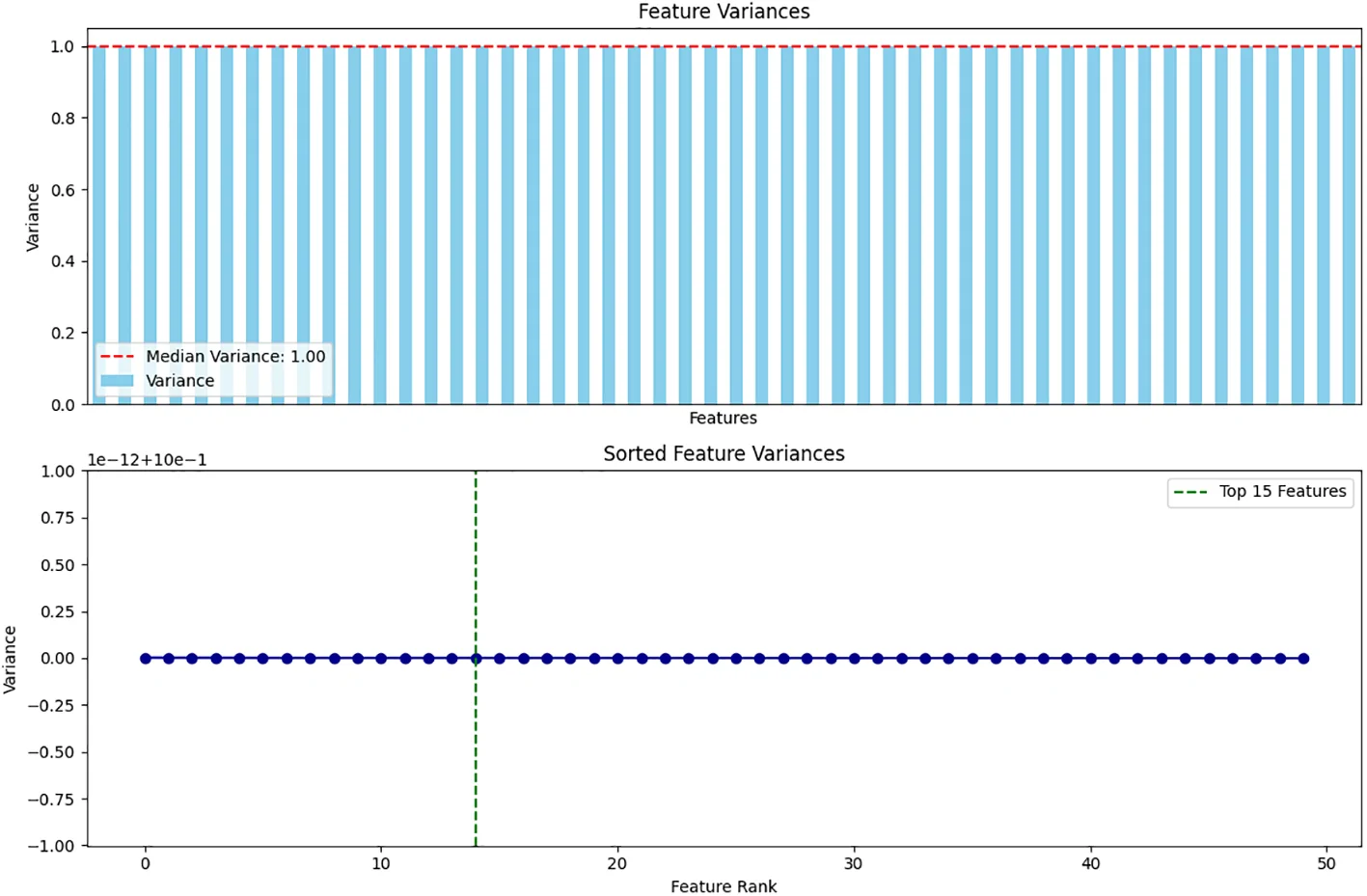
Highly Variable Feature Selection (HVGS) results. Variance of each standardized feature ranked in descending order. The top features with highest inter-subject variability included urbanization level (URBAN), education level, near visual acuity of the left eye (OSNVA), spherical equivalent of the left eye (OSSE), and high myopia (HM), all with variances approaching 1.0 after standardization.

Variance-based feature selection was applied to identified the most variable features across the corhot (**Figure 6**). The top features included urbanization level (URBAN), education, OSNVA, OSSE, and HM (all exhibiting variances approximating 1.0 after standardization).

### 3.5 Nonparametric correlation analysis

**Figure 7:**
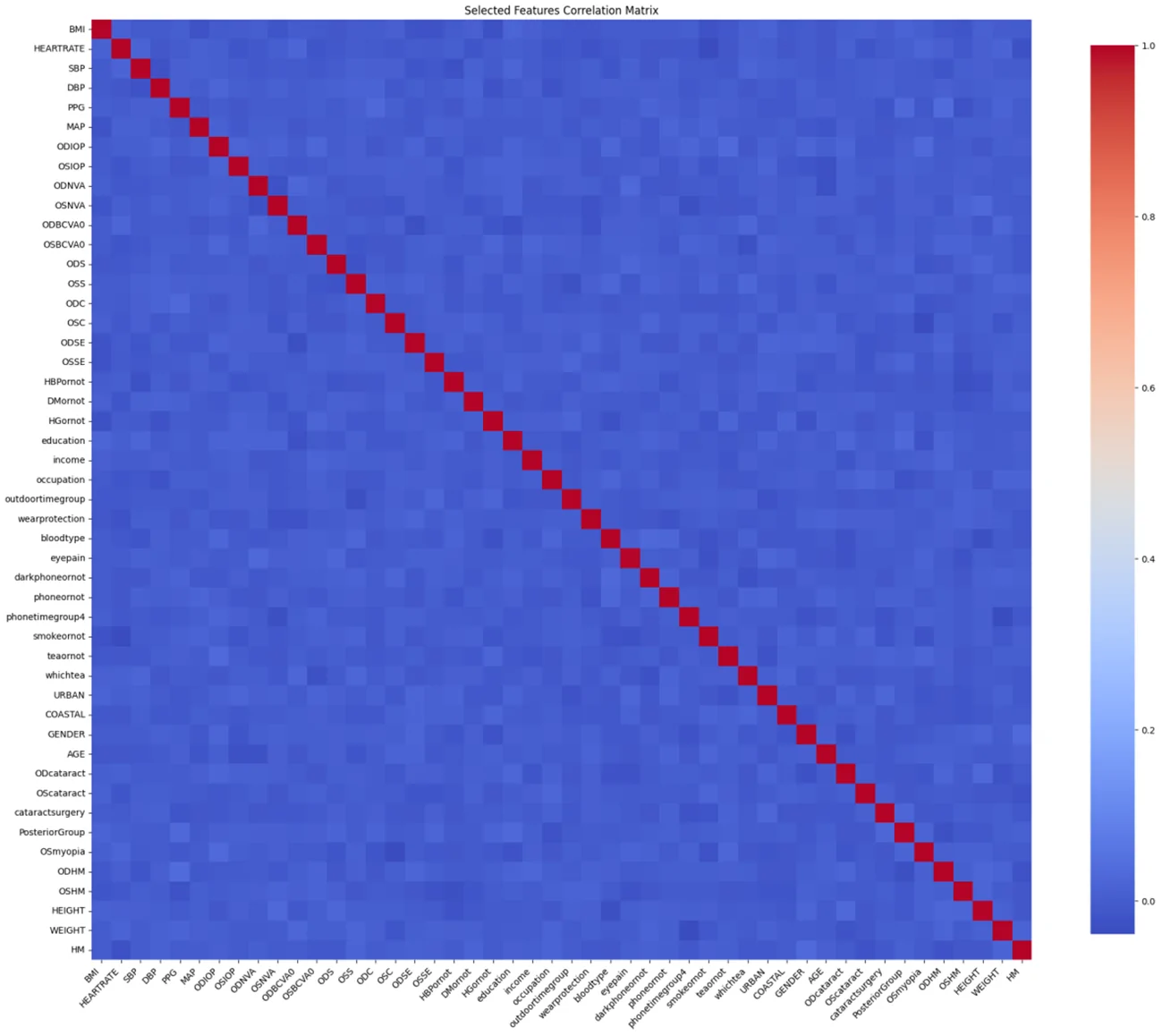
Correlation matrix of selected features (Spearman’s rank correlation). Heatmap showing pairwise correlation coefficients between key clinical variables and ocular parameters. Significant correlations (p < 0.05 after Benjamini-Hochberg FDR correction) are indicated by asterisks. Notable correlations include BMI with DBP (ρ = 0.177, p = 0.079) and BMI with HM (ρ = -0.128, p = 0.204).

Spearman’s rank correlation analysis was conducted between all features and key outcomes (**Figure 7**). BMI, for instance, showed weak but notable correlations with DBP (ρ = 0.177, p = 0.079) and HM (ρ = -0.128, p = 0.204).

### 3.6 Compared feature rankings across methods

**Figure 8:**
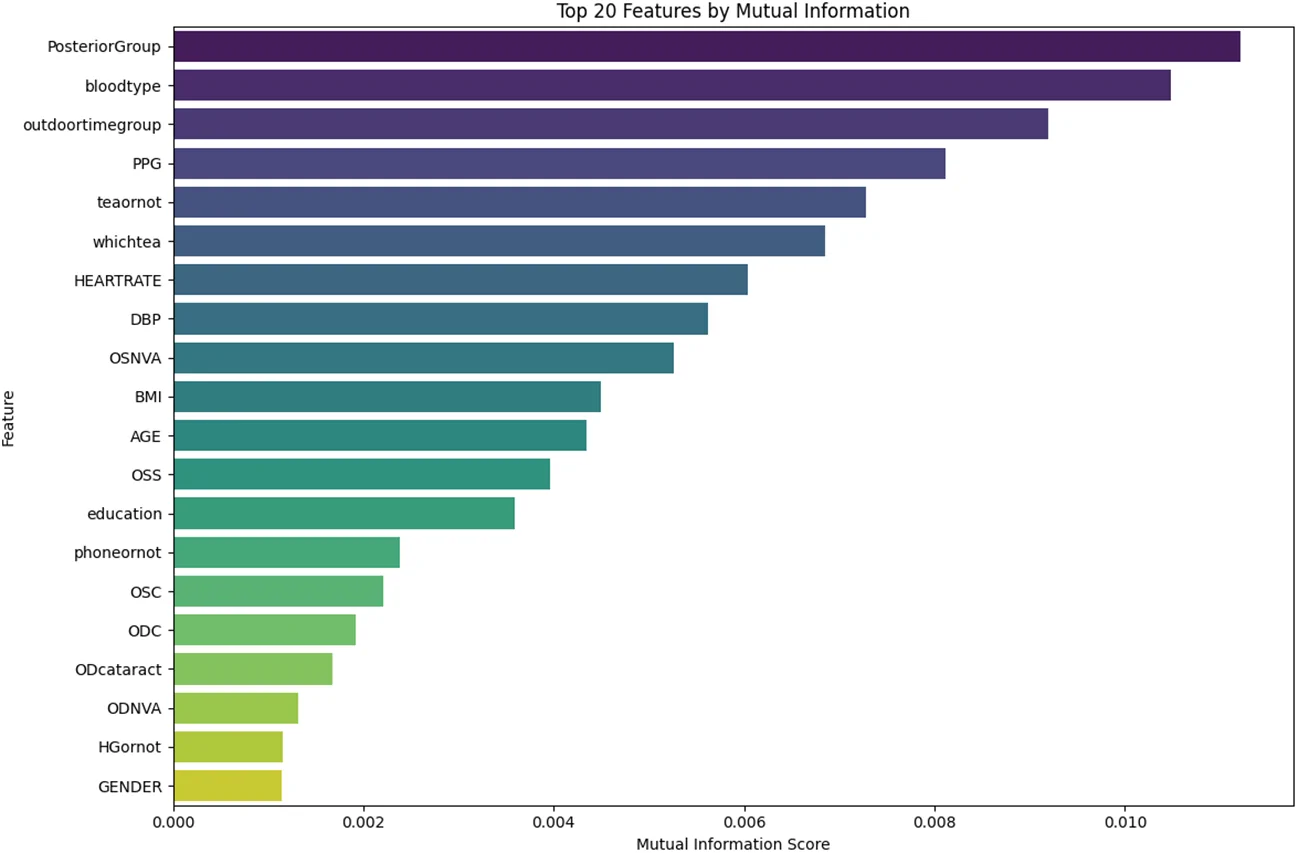
Mutual information scores distribution. Rank correlation analysis comparing feature importance rankings across different methodological approaches (SHAP, Factor Analysis, Highly Variable Feature Selection, and Spearman correlation). Moderate agreement (Spearman's ρ between 0.4–0.6) was observed for top-ranked features, supporting the stability of feature importance rankings.

We computed rank correlation coefficients between SHAP-derived importance and the rankings generated by FA, HVGS, and Spearman correlation (**Figure 8**). Moderate agreement was observed across methods (e.g., Spearman’s ρ between 0.4–0.6 for top-ranked features), supporting the stability of feature importance for variables such as bloodtype, after-cataract findings (PosteriorGroup), and DBP.

### 3.7 Stability test comparison

The stability of feature importance rankings was rigorously evaluated by comparing the outputs of SHAP, Factor Analysis (FA), Highly Variable Feature Selection (HVGS), and Spearman correlation, with the consolidated results presented in **Table 2**. The values in the SHAP column indicate the feature’s rank order of importance, where 1 represents the highest mean absolute SHAP value (most important) and 51 represents the lowest (least important). For example, PPG ranked 3rd overall, with a mean absolute SHAP value of 0.21. This cross-method comparison revealed a strong consensus for several key predictors, underscoring their robustness. Notably, the features PPG, OSNVA, Education, and OSSE were all identified as top contributors by at least two of the three primary machine learning methodologies (SHAP, FA, HVGS), earning a “High” consistency rating. This convergence of evidence from distinct analytical frameworks significantly strengthens the case for their critical role in DR prediction. Furthermore, the non-targeted Spearman correlation analysis provided independent validation, confirming the importance of features previously highlighted in the model-based comparisons, such as bloodtype and PosteriorGroup. The analysis also highlighted the complementary nature of different methods: while SHAP prioritized clinical metrics like HEARTRATE and DBP for their predictive power within the model, HVGS effectively captured demographic variability (e.g., URBAN), and FA grouped underlying ocular biometric constructs (e.g., ODmyopia, OSmyopia). The moderate to high agreement observed across these diverse techniques mitigates concerns of model-specific biases and confirms that the identified core feature set represents stable and reliable determinants of DR risk.

**Table 2:** Stability analysis of feature importance rankings across different methodologies.

Feature	Supervised model (SHAP)	Unsupervised model 1 (FA)	Unsupervised model 2 (HVGS)	Non-targeted model (spearman)	Cross-method consistency
PPG	3	–	Included (Top Feature)	–	High (Agreement in 2/3 ML methods)
OSNVA	4	–	Included (Top Feature)	–	High (Agreement in 2/3 ML methods)
Education	12	–	Included (Top Feature)	–	High (Agreement in 2/3 ML methods)
OSSE	–	Factor 2 (loading = 0.107)	Included (Top Feature)	–	High (Agreement in 2/3 ML methods)
DBP	11	–	–	Notable (with BMI)	Moderate
BMI	16	–	–	Notable (with DBP & HM)	Moderate
HM	–	–	Included (Top Feature)	Notable (with BMI)	Moderate
bloodtype	–	–	–	Consistently Ranked High	Confirmed by Correlation
PosteriorGroup	–	–	–	Consistently Ranked High	Confirmed by Correlation

## 4 Discussion

### 4.1 Comparison and optimization of model performance

Recent years have witnessed growing research efforts devoted to developing machine learning-based predictive models for DR (8). Several prior studies have employed various machine learning algorithms for DR prediction (7, 9–13), though only a limited number have reported AUC values exceeding 0.75 (7).

In the present study, the SVC model achieved outstanding performance with an accuracy of 93.0% and an AUC of 0.99. This finding aligns with previous research highlighting the robustness of ensemble methods, particularly SVC, in handling complex medical datasets with multiple features (13). The model’s capability to manage high-dimensional data while maintaining resistance to overfitting positions it as a strong candidate for DR risk prediction.

Comprehensive evaluation of SVC models requires consideration of multiple metrics and methodologies to ensure objectively assessment. In this study, we employed confusion matrix analysis and ROC curves to conduct detailed performance evaluation. The ROC curve exhibited a steep ascent, indicating high accuracy in predicting positive cases while maintaining a low false positive rate. The model achieved a recall of 97.14%, accuracy of 93.0%, and F1-score of 92.83%, with a high AUC value further confirming its effectiveness in distinguishing between positive and negative samples.

Our SVC model, which integrated 51 multimodal features encompassing biochemical, ocular, and demographic parameters, demonstrated superior performance compared to image-only deep learning models such as ResNet (AUC = 0.85) (14) and transformer-based architectures (AUC = 0.88) (15). However, we acknowledge that this comparison is not directly equivalent due to fundamental differences in input data modality (tabular features vs. retinal images), task definitions, and dataset characteristics. Although these results corroborate recent findings by Ting et al. (16) emphasizing the value of combining systemic and retinal data for DR risk stratification,while the model’s computational efficiency (FLOPs=2.1B) trails behind lightweight architectures like MobileNet (FLOPs=0.6B) (17), indicating a need for optimization to facilitate real-world deployment. Direct head-to-head comparison of structured-data machine learning and image-based deep learning on the same dataset represents a valuable direction for future research. Additionally, applying deep learning architectures specifically designed for tabular data (such as TabNet or FT-Transformer) to our dataset could provide more meaningful comparisons and is an avenue we plan to explore in subsequent studies.

Potential approaches for enhancing model performance include adjusting hyperparameters such as the number of trees, tree depth, and minimum samples per leaf. Future investigations could also explore more advanced ensemble methods, including gradient boosting machines (e.g., XGBoost, LightGBM) and stacking models, which have shown promise in other medical prediction tasks (14, 18).

Moreover, the integration of deep learning approaches, particularly convolutional neural networks (CNNs), for direct retinal image analysis represents a promising direction. CNNs have been successfully implemented in DR detection systems, achieving state-of-the-art performance in automated grading applications (14, 19). The combination of traditional machine learning models with deep learning methodologies may potentially yield enhanced predictive accuracy while providing more comprehensive insights into DR risk factors.

### 4.2 Clinical implications of feature importance

DR is influenced by a diverse of physiological and lifestyle-related risk factors. Well-established determinants include chronic hyperglycemia, hypertension, and dyslipidemia, longer duration of diabetes, poor glycemic control (high HbA1c levels), and obesity (20–23). Additional contributing factors such as smoking, genetic susceptibility, and coexisting cardiovascular diseases have also been shown to elevate DR risk (24–26). Furthermore, age and pregnancy represent significant risk modifiers, particularly among individuals with diabetes (27, 28). These risk elements have been extensively studied across diverse patient populations and clinical settings (29, 30).

A key advantage of the random forest model lies in its capacity to perform robust feature importance analysis. By quantifying the reduction in impurity or accuracy loss attributable to each feature during the decision the tree-building process, the model effectively measures the relative contribution of individual variable to predictive performance.

Our SHAP-based feature importance analysis revealed diabetes history, age, PPG, OSNVA, and height as the most influential predictors of DR. These findings align closely with established clinical knowledge, as both diabetes duration and advanced age are widely recognized as prominent risk factors for DR (31). Notably, the combination of systemic factors such as PPG—an indicator of arterial stiffness linked to cardiovascular risk in diabetic patients (3)—and ocular parameters like NVA, which may reflect early retinal changes, collectively explained 67% of the model’s decision variance. This pattern is consistent with the understood pathophysiological mechanisms underlying DR progression (21), underscoring the complementary value of systemic health indicators and ocular functional metrics in comprehensive DR risk assessment.

These insights not only enhance the model’s predictive accuracy but also provide clinically actionable information to support early intervention strategies. For instance, patients presenting with prolonged diabetes history, elevated PPG, or impaired NVA could be prioritized for more frequent screenings or targeted interventions. Such an approach resonates with the growing emphasis on personalized medicine, wherein risk stratification tools play an increasingly vital role in tailoring prevention and treatment strategies to individual patient profiles (8).

Height emerged as a notable predictor in our SHAP analysis. Although the biological mechanism linking height directly to DR pathogenesis is not fully established, height may serve as a surrogate marker for early-life nutritional status, developmental factors, or genetic determinants that influence both stature and susceptibility to metabolic diseases. Alternatively, height may capture residual confounding related to body composition or hemodynamic factors. Future studies incorporating genetic and longitudinal data are warranted to elucidate the mechanistic pathways underlying this association.

### 4.3 Practical applications and challenges

Although the results of this study are encouraging, the translation of machine learning models into routine clinical practice presents several challenges. First, successful integration into healthcare workflows requires seamless interoperability with existing electronic health records (EHRs) and clinical databases. Ensuring real-time data accessibility and timely model updates is essential to maintain prediction accuracy and clinical relevance (9). Second, the acceptance of machine learning-driven predictions among both clinicians and patients remain considerable barrier. Clinicians may exhibit reluctance to trust “black-box” models, especially in high-stakes clinical decision-making. Improving model interpretability through techniques such as SHAP values and local interpretable model-agnostic explanations (LIME) can enhance transparency and foster trust, thereby facilitating broader adoption (32).

A further challenge lies in the cost-effectiveness of model deployment, particularly in resource-limited settings. While machine learning models hold potential to alleviate healthcare burdens by automating screening, the initial investments required for infrastructure, standardized data collection, and model training can be substantial. Future research should incorporate rigorous cost-benefit analyses to evaluate the economic viability of implementing machine learning-based DR prediction systems across diverse healthcare environments (33).

### 4.4 Future research directions

Future research should focus on several key areas to advance DR risk prediction and clinical management:

#### 4.4.1 Multi-center validation

External validation across diverse populations and geographic regions is essential to verify model generalizability. Variations in demographics, healthcare access, and disease prevalence may significantly impact model performance. Multi-center collaborative studies can help identify and mitigate these discrepancies, thereby enhancing model robustness and widely applicability (34).

#### 4.4.2 Deep learning integration

While traditional machine learning models have shown considerable potential, deep learning approaches—particularly CNNs—offer enhanced capabilities for retinal image analysis. These models can automatically extract discriminative features from fundus photographs, potentially improving DR detection and grading accuracy. Future work should explore hybrid framework that combine deep learning with traditional machine learning to leverage their complementary strengths (35).

#### 4.4.3 Real-time clinical decision support

The development of real-time prediction systems integrated into routine clinical workflows represents a critical advancement. Such systems could deliver instant risk assessments during patient consultations, enabling timely interventions. Successful implementation will require robust computational infrastructure for efficient data processing and model inference, coupled with intuitive user interfaces to promote clinician adoption (36).

#### 4.4.4 Personalized intervention strategies

The application of machine learning to develop personalized prevention and treatment strategies presents a promising avenue research. Models could identify patient subgroups at elevated risk for DR progression based on unique genetic, clinical, or comorbidity profiles, enabling tailored therapeutic approaches and personalized monitoring schedules (37).

#### 4.4.5 Ethical and regulatory frameworks

As machine learning becomes increasingly embedded in clinical practice, addressing associated ethical and regulatory concerns will be essential. Issues such as data privacy, algorithmic bias, health equity, and informed consent require comprehensive solutions. Collaborative efforts among researchers, clinicians, and regulatory bodies will be crucial to establish guidelines ensuring the responsible and equitable deployment of AI in healthcare (38).

#### 4.4.6 Prediction of age at DR onset

Another promising direction is the prediction of age at DR onset—a clinically valuable target for personalized screening intervals. While our current cross-sectional study identifies baseline risk factors, predicting the timing of DR onset requires longitudinal data and time-to-event modeling approaches such as survival analysis (e.g., Cox proportional hazards, Random Survival Forests, or DeepSurv). These methods can accommodate right-censored data and provide estimates of the probability of remaining DR-free over time. In future work, we plan to incorporate repeated measurements from ongoing cohort follow-up to develop dynamic prediction models that estimate individualized DR onset trajectories. Such models could enable personalized screening schedules—for example, recommending more frequent screenings for individuals predicted to develop DR within a shorter time horizon—thereby optimizing resource allocation and potentially reducing the burden of vision loss in diabetic populations.

### 4.5 Limitations and methodological considerations for feature interpretation

While our model demonstrated strong predictive performance, several important limitations regarding feature interpretability warrant consideration. Firstly, our study compares the performance of multiple machine learning models for DR prediction using epidemiological data, it does not provide deeper mechanistic insights into the underlying disease pathways or deliver highly specific clinical action plans. The absence of molecular, genetic, or multi-omics data limits our ability to contextualize feature importance within biological pathways. Future research incorporating multimodal data sources is warranted to elucidate the functional roles of identified predictors and to translate model outputs into actionable clinical guidance. In addition, although internal validation results are promising, the lack of external validation on independent datasets prevents definitive conclusions regarding model performance across diverse populations and clinical settings. Should suitable datasets become available, comprehensive external validation will be essential to further assess model robustness and generalizability. Furthermore, we acknowledge that variance alone is not the most robust feature selection criterion. HVGS was employed as a supplementary, unsupervised method to identify features with high natural variability across the population, serving as a data-driven sanity check rather than a primary feature selection tool. The primary feature importance analysis was conducted using SHAP values (supervised, model-based), which directly measure each feature’s contribution to DR prediction. We also acknowledge that alternative methods such as forward selection or recursive feature elimination (RFE) could be considered for feature selection; however, these were beyond the scope of the current study, which prioritized predictive performance using the full set of clinically available features.

In summary, this study indicate that the SVC model achieves strong classification performance in DR prediction, while feature importance analysis provides valuable insights for model optimization. Future work will fucus on evaluating SVC model performance across diverse datasets and exploring the application of additional machine learning algorithms in ophthalmic classification tasks.

## Data Availability

The original contributions presented in the study are included in the article/**Supplementary Material**. Further inquiries can be directed to the corresponding author.
