# Supplementary material for: Machine learning models for risk prediction of diabetic retinopathy in Fujian eye study
Source: Front Endocrinol (Lausanne). 2026 Aug 6;17:1834380. doi: 10.3389/fendo.2026.1834380 (PMC13489881; doi:10.3389/fendo.2026.1834380)
Supplement: Supplementary file 1 [file DataSheet2.docx]

```
#激活虚拟环境study
conda activate study


#运行jupyter软件
jupyter lab


#粘贴地址 http://localhost:8888/lab/tree/Desktop/12_AI%E8%BE%85%E5%8A%A9%E4%B8%B4%E5%BA%8A%E6%95%B0%E6%8D%AE%E6%8C%96%E6%8E%98/diabetes/eda-and-modelling-for-diabetes.ipynb


#安装软件包jupyter\numpy\pandas\matplotlib\seaborn(包名)
pip install numpy -i https://mirrors.aliyun.com/pypi/simple/

pip install pandas -i https://mirrors.aliyun.com/pypi/simple/

pip install matplotlib -i https://mirrors.aliyun.com/pypi/simple/

pip install seaborn -i https://mirrors.aliyun.com/pypi/simple/

pip install xlrd -i https://mirrors.aliyun.com/pypi/simple/

pip install openpyxl -i https://mirrors.aliyun.com/pypi/simple/

pip install scikit-learn -i https://mirrors.aliyun.com/pypi/simple/

pip install ydata_profiling -i https://mirrors.aliyun.com/pypi/simple/

pip install lightgbm -i https://pypi.tuna.tsinghua.edu.cn/simple

pip install xgboost -i https://pypi.tuna.tsinghua.edu.cn/simple


#jupyter中导入工具包
import numpy as np ##数值计算框架－－软件包
import pandas as pd ## 表格类文件处理工具
import matplotlib.pyplot as plt #绘图工具
import warnings
import seaborn as sns
warnings.filterwarnings('ignore')


#停止jupyter软件
两次control C


#每次下载一个新的数据程序包，都要重新启动jupyter软件


#1.数据读入
import pandas as pd
df = pd.read_excel('/Users/lotus/desktop/2024.07.31-FJES数据GPT用-2补充缺失值-DR.xlsx', engine='openpyxl')


#2数据校验
#2.1观察
df.info()


#2.2描述分析
#2.2.1变量不多时用
df.describe()


#2.2.2变量多时，观察每个变量的最小值（最大值、均值、标准差。。。。
for col in df.columns:
    print("{}最小值是 {}".format(col, df[col].min()))


#2.3缺失值校验
df.isnull().sum()

#自动化数据探索分析软件包
# from ydata_profiling import ProfileReport
# df_anasys = ProfileReport(df)
# # 生成HTML报告
# report = ProfileReport(df)
# report.to_file("report.html")


#3.1数据清洗
import numpy as np


df.min(numeric_only=True)


df.min()


df.isnull().sum()


没有缺失值，不需要处理
```

```
#模型构建
（y为DR）

#数据集划分及预处理
import pandas as pd
df = pd.read_excel('/Users/lotus/desktop/2024.07.31-FJES数据GPT用-2补充缺失值-DR.xlsx', sheet_name='Sheet1')


from sklearn.model_selection import train_test_split

# Assuming 'df' is your DataFrame and 'target' is the column you want to predict
X = df.drop('DR', axis=1)  # Features
y = df['DR']  # Target variable

# Split the data into training and testing sets
X_train, X_test, y_train, y_test = train_test_split(X, y, test_size=0.3, random_state=20)


X_train.shape
(5747, 51)


X_test.shape
(2464, 51)


#数据标准化处理


# 导入必要的库
from sklearn.neighbors import KNeighborsClassifier
from sklearn.model_selection import GridSearchCV
from sklearn.preprocessing import StandardScaler
from sklearn.metrics import accuracy_score
import pandas as pd

# 假设你已经有了以下数据集
# X_train, y_train = ...  # 训练数据
# X_test = ...           # 测试数据

# 确认你的数据已经被适当地预处理
# 这里我们添加了一个简单的标准化步骤
scaler = StandardScaler()  # 导入StandardScaler类
X_train_scaled = scaler.fit_transform(X_train)
X_test_scaled = scaler.transform(X_test)
sc = StandardScaler()
X_train =  pd.DataFrame(sc.fit_transform(X_train),
        columns=['BMI', 'HEARTRATE', 'SBP', 'DBP', 'PPG', 'MAP', 'ODIOP', 'OSIOP', 'ODNVA', 'OSNVA', 'ODBCVA0', 'OSBCVA0', 'ODS', 'OSS', 'ODC', 'OSC', 'ODSE', 'OSSE', 'HBPornot', 'DMornot', 'HGornot', 'education', 'income', 'occupation', 'outdoortimegroup', 'wearprotection', 'bloodtype', 'eyediscomfort', 'eyepain', 'darkphoneornot', 'phoneornot', 'phonetimegroup4', 'smokeornot', 'drinkornot', 'teaornot', 'whichtea', 'URBAN', 'COASTAL', 'GENDER', 'AGE', 'ODcataract', 'OScataract', 'ODmyopia', 'cataractsurgery', 'PosteriorGroup', 'OSmyopia', 'ODHM', 'OSHM', 'HEIGHT', 'WEIGHT', 'HM'])
X_test =  pd.DataFrame(sc.fit_transform(X_test),
        columns=['BMI', 'HEARTRATE', 'SBP', 'DBP', 'PPG', 'MAP', 'ODIOP', 'OSIOP', 'ODNVA', 'OSNVA', 'ODBCVA0', 'OSBCVA0', 'ODS', 'OSS', 'ODC', 'OSC', 'ODSE', 'OSSE', 'HBPornot', 'DMornot', 'HGornot', 'education', 'income', 'occupation', 'outdoortimegroup', 'wearprotection', 'bloodtype', 'eyediscomfort', 'eyepain', 'darkphoneornot', 'phoneornot', 'phonetimegroup4', 'smokeornot', 'drinkornot', 'teaornot', 'whichtea', 'URBAN', 'COASTAL', 'GENDER', 'AGE', 'ODcataract', 'OScataract', 'ODmyopia', 'cataractsurgery', 'PosteriorGroup', 'OSmyopia', 'ODHM', 'OSHM', 'HEIGHT', 'WEIGHT', 'HM'])


X_train


#预测模型构建

from sklearn.linear_model import LogisticRegression #逻辑回归--分类
from sklearn.neighbors import KNeighborsClassifier #最近邻
from sklearn.svm import SVC #支持向
from sklearn.tree import DecisionTreeClassifier#决策树
from sklearn.ensemble import RandomForestClassifier#随机森林
from sklearn.metrics import confusion_matrix #评价指标（模型)
from sklearn.model_selection import GridSearchCV #网络搜索调参
from lightgbm import LGBMClassifier #一个集成学习算法
import xgboost as xgb #集成学习算法


#第一个模型：逻辑回归模型
from sklearn.linear_model import LogisticRegression

# 创建逻辑回归模型的一个实例
log_model = LogisticRegression()

# 假设 X_train 和 y_train 已经定义，并且是你的训练数据和标签
log_model.fit(X_train, y_train) #模型构建--函数的拟合

log_predict = log_model.predict(X_test)


# 假设 X_test 和 y_test 是你的测试数据和标签
log_score = log_model.score(X_test, y_test)
print(f"模型的准确率是: {log_score}")


log_score


#第二个模型：KNN最近模型
# 导入必要的库
from sklearn.neighbors import KNeighborsClassifier
from sklearn.model_selection import GridSearchCV
from sklearn.preprocessing import StandardScaler
from sklearn.metrics import accuracy_score

# 假设你已经有了以下数据集
# X_train, y_train = ...  # 训练数据
# X_test = ...           # 测试数据

# 确认你的数据已经被适当地预处理
# 这里我们添加了一个简单的标准化步骤
scaler = StandardScaler()
X_train_scaled = scaler.fit_transform(X_train)
X_test_scaled = scaler.transform(X_test)

# 设置KNN模型的参数
knn_params = {
    'n_neighbors': list(range(3, 20, 2)),
    'weights': ['uniform', 'distance'],
    'algorithm': ['auto', 'ball_tree', 'kd_tree', 'brute'],
    'metric': ['euclidean', 'manhattan', 'chebyshev']
}

# 创建一个KNN分类器实例
knn = KNeighborsClassifier()

# 创建GridSearchCV实例
knn_model = GridSearchCV(knn, knn_params, cv=5)

# 训练模型
knn_model.fit(X_train_scaled, y_train)

# 进行预测
knn_predict = knn_model.predict(X_test_scaled)

# 获取最佳交叉验证分数
knn_score = knn_model.best_score_

# 计算预测准确率
accuracy = accuracy_score(y_test, knn_predict)
print(f"Best Cross-Validation Score: {knn_score:.4f}")
print(f"Test Accuracy: {accuracy:.4f}")


knn_score


#第三个模型：支持向量机
# Support Vector Classification(svc)
svc_params = {'C': [0.001, 0.01, 0.1, 1],
              'kernel': [ 'linear' , 'poly' , 'rbf' , 'sigmoid' ]}
svc_model = GridSearchCV(SVC(), svc_params, cv=5) #Tuning the hyper-parameters
svc_model.fit(X_train, y_train)
svc_predict = svc_model.predict(X_test)
svc_score = svc_model.best_score_


svc_score


第四个模型：决策树
# Decision Tree
dt_params = {'criterion' : ['gini', 'entropy'],
              'splitter': ['random', 'best'],
              'max_depth': [3, 5, 7, 9, 11, 13]}
dt_model = GridSearchCV(DecisionTreeClassifier(), dt_params, cv=5) #Tuning the hyper-parameters
dt_model.fit(X_train, y_train)
dt_predict = dt_model.predict(X_test)
dt_score = dt_model.best_score_


dt_score


第五个模型：随机森林
# Random Forest
rf_params = {'criterion' : ['gini', 'entropy'],
             'n_estimators': list(range(5, 26, 5)),
             'max_depth': list(range(3, 20, 2))}
rf_model = GridSearchCV(RandomForestClassifier(), rf_params, cv=5) #Tuning the hyper-parameters
rf_model.fit(X_train, y_train)
rf_predict = rf_model.predict(X_test)
rf_score = rf_model.best_score_


rf_score


第六个模型：lgbboabsost
# 导入必要的库
from lightgbm import LGBMClassifier
from sklearn.model_selection import GridSearchCV
from sklearn.preprocessing import StandardScaler
from sklearn.metrics import accuracy_score
import pandas as pd

# 假设你已经有了以下数据集
# X_train, y_train = ...  # 训练数据
# X_test = ...           # 测试数据

# 确认你的数据已经被适当地预处理
# 这里我们添加了一个简单的标准化步骤
scaler = StandardScaler()  # 导入StandardScaler类
X_train_scaled = scaler.fit_transform(X_train)
X_test_scaled = scaler.transform(X_test)

# 设置LGBM分类器的参数
lgb_params = {
    'n_estimators': [5, 10, 15, 20, 25, 50, 100],
    'learning_rate': [0.01, 0.05, 0.1],
    'num_leaves': [7, 15, 31],
}

# 创建一个LGBM分类器实例
lgb_classifier = LGBMClassifier()  # 导入LGBMClassifier类

# 创建GridSearchCV实例
lgb_model = GridSearchCV(lgb_classifier, lgb_params, cv=5)

# 训练模型
lgb_model.fit(X_train_scaled, y_train)

# 进行预测
lgb_predict = lgb_model.predict(X_test_scaled)

# 获取最佳交叉验证分数
lgb_score = lgb_model.best_score_


lgb_score
```

# 第七个模型：XGBOOST

```
# pip install xgboost -i https://pypi.tuna.tsinghua.edu.cn/simple

# 导入必要的库
from sklearn.model_selection import GridSearchCV
from sklearn.preprocessing import StandardScaler
from sklearn.metrics import accuracy_score
import pandas as pd
from xgboost import XGBClassifier

# 假设你已经有了以下数据集
# X_train, y_train = ...  # 训练数据
# X_test = ...           # 测试数据

# 确认你的数据已经被适当地预处理
# 这里我们添加了一个简单的标准化步骤
scaler = StandardScaler()  # 导入StandardScaler类
X_train_scaled = scaler.fit_transform(X_train)
X_test_scaled = scaler.transform(X_test)

# 设置XGB分类器的参数
xgb_params = {
    'max_depth': [3, 5, 7, 9,10],
    'n_estimators': [1,5, 10, 15, 20],
    'learning_rate': [0.01, 0.05, 0.1],
}

# 创建一个XGB分类器实例
xgb_classifier = XGBClassifier(eval_metric='logloss')  # 导入XGBClassifier类

# 创建GridSearchCV实例
xgb_model = GridSearchCV(xgb_classifier, xgb_params, cv=5)

# 训练模型
xgb_model.fit(X_train_scaled, y_train)

# 进行预测
xgb_predict = xgb_model.predict(X_test_scaled)

# 获取最佳交叉验证分数
xgb_score = xgb_model.best_score_

# 计算预测准确率
accuracy = accuracy_score(y_test, xgb_predict)
print(f"Best Cross-Validation Score: {xgb_score:.2f}")
print(f"Test Accuracy: {accuracy:.2f}")


Best Cross-Validation Score: 0.98
Test Accuracy: 0.98

xgb_score


#模型评估

#单个模型评估
#LightGBM模型
from lightgbm import LGBMClassifier
from sklearn.model_selection import GridSearchCV #网络搜索调参
from sklearn.metrics import classification_report

# 假设你已经有了以下数据集
# X_train, y_train = ...  # 训练数据
# X_test, y_test = ...    # 测试数据

# 创建分类器实例
lgb_model = LGBMClassifier()

# 训练模型
lgb_model.fit(X_train, y_train)

# 使用训练好的模型进行预测
lgb_model_predict = lgb_model.predict(X_test)

# 打印分类报告
print(classification_report(lgb_model_predict, y_test))


#多个模型accuracy比较

import pandas as pd
import seaborn as sns
import matplotlib.pyplot as plt

# 定义模型名称列表
models = ['Logistic Regression', 'KNN', 'SVC', 'Decision Tree', 'Random Forest', 'LightGBM', 'XGBOOST']

# 假设你已经有了一个包含模型评分的列表
scores = [0.975649, 0.977206, 0.977932, 0.977042, 0.977236, 0.977554, 0.977032]  # 示例分数


# 使用模型名称和评分创建一个DataFrame
score_table = pd.DataFrame({'Model': models, 'Score': scores})

# 排序DataFrame
score_table = score_table.sort_values(by='Score', ascending=False)

# 打印排序后的DataFrame
print(score_table)


# 确保您的DataFrame已经正确创建
# score_table = pd.DataFrame({'Model': models, 'Score': scores})

# 创建条形图
sns.barplot(x='Score', y='Model', hue='Model', data=score_table, palette='inferno')

# 显示图表
plt.show()


#多个模型综合性能比较

import pandas as pd
import numpy as np
from sklearn.model_selection import train_test_split
from sklearn.metrics import accuracy_score, precision_score, recall_score, f1_score, classification_report
from sklearn.ensemble import RandomForestClassifier, GradientBoostingClassifier, AdaBoostClassifier
from sklearn.linear_model import LogisticRegression
from sklearn.svm import SVC
from sklearn.neighbors import KNeighborsClassifier
from sklearn.tree import DecisionTreeClassifier
from sklearn.datasets import make_classification

# 创建示例数据集
X, y = make_classification(n_samples=1000, n_features=20, n_informative=15,
                           n_redundant=5, n_classes=2, random_state=42)
X_train, X_test, y_train, y_test = train_test_split(X, y, test_size=0.3, random_state=42)

# 定义模型
models = {
    'Random Forest': RandomForestClassifier(n_estimators=100, random_state=42),
    'LightGBM': GradientBoostingClassifier(n_estimators=100, random_state=42),
    'XGBoost': AdaBoostClassifier(n_estimators=100, random_state=42),
    'Logistic Regression': LogisticRegression(max_iter=1000, random_state=42),
    'SVC': SVC(probability=True, random_state=42),
    'K-Nearest Neighbors': KNeighborsClassifier(),
    'Decision Tree': DecisionTreeClassifier(random_state=42)
}

# 存储结果的列表
results = []

# 训练和评估每个模型
for name, model in models.items():
    # 训练模型
    model.fit(X_train, y_train)
    # 预测
    y_pred = model.predict(X_test)

    # 计算指标
    accuracy = accuracy_score(y_test, y_pred)
    precision = precision_score(y_test, y_pred)
    recall = recall_score(y_test, y_pred)
    f1 = f1_score(y_test, y_pred)

    # 获取支持度 (每个类的样本数)
    support_0 = len(y_test[y_test == 0])
    support_1 = len(y_test[y_test == 1])

    # 存储结果
    results.append({
        'Model': name,
        'Accuracy': accuracy,
        'Precision': precision,
        'Recall': recall,
        'F1-Score': f1,
        'Support (Class 0)': support_0,
        'Support (Class 1)': support_1
    })

# 创建结果DataFrame
results_df = pd.DataFrame(results)

# 设置模型名为索引
results_df.set_index('Model', inplace=True)

# 打印详细结果
print("模型性能比较:")
print("=" * 80)
print(results_df.round(4))

# 打印每个模型的详细分类报告
print("\n\n每个模型的详细分类报告:")
print("=" * 80)
for name, model in models.items():
    model.fit(X_train, y_train)
    y_pred = model.predict(X_test)
    print(f"\n{name}分类报告:")
    print("-" * 40)
    print(classification_report(y_test, y_pred, digits=4))
```

```
#混淆矩阵

import matplotlib.pyplot as plt
import seaborn as sns
from sklearn.metrics import confusion_matrix, ConfusionMatrixDisplay
import numpy as np

# 假设我们已经有了测试集的真实标签y_test和最佳模型的预测结果y_pred_best
# 这里我们假设Random Forest是最佳模型，实际应用中应根据评估结果选择最佳模型

# 找到最佳模型（以F1分数为例）
best_model_name = results_df['F1-Score'].idxmax()
best_model = models[best_model_name]

# 重新训练最佳模型（如果需要）
best_model.fit(X_train, y_train)
y_pred_best = best_model.predict(X_test)

# 计算混淆矩阵
cm = confusion_matrix(y_test, y_pred_best)

# 绘制混淆矩阵
plt.figure(figsize=(8, 6))
sns.heatmap(cm, annot=True, fmt='d', cmap='Blues',
            xticklabels=['Predicted Negative', 'Predicted Positive'],
            yticklabels=['Actual Negative', 'Actual Positive'])
plt.title(f'Confusion Matrix for {best_model_name}')
plt.ylabel('True Label')
plt.xlabel('Predicted Label')
plt.show()

# 打印详细指标
tn, fp, fn, tp = cm.ravel()

print(f"最佳模型: {best_model_name}")
print("=" * 50)
print(f"真阴性 (True Negatives, TN): {tn}")
print(f"假阳性 (False Positives, FP): {fp}")
print(f"假阴性 (False Negatives, FN): {fn}")
print(f"真阳性 (True Positives, TP): {tp}")
print("=" * 50)
print(f"准确率 (Accuracy): {(tp + tn) / (tp + tn + fp + fn):.4f}")
print(f"精确率 (Precision): {tp / (tp + fp):.4f}")
print(f"召回率 (Recall/Sensitivity): {tp / (tp + fn):.4f}")
print(f"特异度 (Specificity): {tn / (tn + fp):.4f}")
print(f"F1分数: {2 * tp / (2 * tp + fp + fn):.4f}")
```

```
#多模型比较
import warnings
warnings.filterwarnings('ignore')  # 忽略警告

import numpy as np
import pandas as pd
import matplotlib.pyplot as plt
import seaborn as sns
from sklearn.datasets import make_classification
from sklearn.model_selection import train_test_split, GridSearchCV
from sklearn.metrics import accuracy_score, classification_report, confusion_matrix, roc_curve, auc
from sklearn.preprocessing import StandardScaler, label_binarize

# 导入其他机器学习模型
from sklearn.ensemble import RandomForestClassifier, GradientBoostingClassifier, AdaBoostClassifier, ExtraTreesClassifier
from sklearn.linear_model import LogisticRegression
from sklearn.svm import SVC
from sklearn.neighbors import KNeighborsClassifier
from sklearn.tree import DecisionTreeClassifier
from sklearn.naive_bayes import GaussianNB
from sklearn.discriminant_analysis import LinearDiscriminantAnalysis, QuadraticDiscriminantAnalysis

# 设置随机种子
np.random.seed(42)

# 创建模拟数据
print("创建模拟数据...")
X, y = make_classification(n_samples=1000, n_features=20, n_informative=10,
                           n_redundant=5, n_classes=2, random_state=42)

# 数据标准化
scaler = StandardScaler()
X_scaled = scaler.fit_transform(X)

# 分割数据
X_train, X_test, y_train, y_test = train_test_split(X_scaled, y, test_size=0.3, random_state=42)

# 初始化模型字典
models = {
    'Random Forest': RandomForestClassifier(n_estimators=100, random_state=42),
    'LightGBM': GradientBoostingClassifier(n_estimators=100, random_state=42),
    'XGBoost': AdaBoostClassifier(n_estimators=100, random_state=42),
    'Logistic Regression': LogisticRegression(max_iter=1000, random_state=42),
    'SVC': SVC(probability=True, random_state=42),
    'K-Nearest Neighbors': KNeighborsClassifier(),
    'Decision Tree': DecisionTreeClassifier(random_state=42),
}

# 训练和评估模型
results = []
print("\n训练和评估模型...")
for name, model in models.items():
    print(f"训练 {name}...")
    model.fit(X_train, y_train)
    y_pred = model.predict(X_test)
    y_pred_proba = model.predict_proba(X_test)[:, 1] if hasattr(model, "predict_proba") else None

    accuracy = accuracy_score(y_test, y_pred)

    results.append({
        'Model': name,
        'Accuracy': accuracy,
        'Model_Obj': model,
        'Y_Pred': y_pred,
        'Y_Pred_Proba': y_pred_proba
    })

    print(f"  {name} 准确率: {accuracy:.4f}")

# 创建结果数据框
results_df = pd.DataFrame(results)
print("\n模型性能比较:")
print(results_df[['Model', 'Accuracy']].sort_values('Accuracy', ascending=False))

# 可视化结果
plt.figure(figsize=(14, 8))
bars = plt.bar(results_df['Model'], results_df['Accuracy'])
plt.title('模型准确率比较', fontsize=16)
plt.xlabel('模型', fontsize=12)
plt.ylabel('准确率', fontsize=12)
plt.xticks(rotation=45, ha='right')
plt.ylim(0, 1)
plt.grid(axis='y', linestyle='--', alpha=0.7)

# 在柱状图上添加数值标签
for bar in bars:
    height = bar.get_height()
    plt.text(bar.get_x() + bar.get_width()/2., height + 0.01,
             f'{height:.3f}', ha='center', va='bottom')

plt.tight_layout()
plt.savefig('model_comparison.png', dpi=300, bbox_inches='tight')
print("\n可视化结果已保存为 'model_comparison.png'")
plt.show()

# 显示最佳模型的详细报告
best_model_idx = results_df['Accuracy'].idxmax()
best_model_name = results_df.loc[best_model_idx, 'Model']
best_model = results_df.loc[best_model_idx, 'Model_Obj']
y_pred = results_df.loc[best_model_idx, 'Y_Pred']

print(f"\n最佳模型: {best_model_name}")
print("分类报告:")
print(classification_report(y_test, y_pred))

print("混淆矩阵:")
cm = confusion_matrix(y_test, y_pred)
cm_df = pd.DataFrame(cm,
                     index=['True Negative', 'True Positive'],
                     columns=['Predicted Negative', 'Predicted Positive'])
print(cm_df)

# 特征重要性（如果可用）
if hasattr(best_model, 'feature_importances_'):
    print(f"\n{best_model_name} 特征重要性 (前10个):")
    feature_importance = pd.DataFrame({
        'feature': range(X.shape[1]),
        'importance': best_model.feature_importances_
    }).sort_values('importance', ascending=False).head(10)
    print(feature_importance)

# ROC 曲线（如果模型支持概率预测）
plt.figure(figsize=(10, 8))
for i, result in enumerate(results):
    if result['Y_Pred_Proba'] is not None:
        fpr, tpr, _ = roc_curve(y_test, result['Y_Pred_Proba'])
        roc_auc = auc(fpr, tpr)
        plt.plot(fpr, tpr, label=f'{result["Model"]} (AUC = {roc_auc:.2f})')

plt.plot([0, 1], [0, 1], 'k--')
plt.xlim([0.0, 1.0])
plt.ylim([0.0, 1.05])
plt.xlabel('False Positive Rate')
plt.ylabel('True Positive Rate')
plt.title('ROC 曲线比较')
plt.legend(loc="lower right")
plt.grid(True, alpha=0.3)
plt.savefig('roc_curves.png', dpi=300, bbox_inches='tight')
plt.show()
print("ROC 曲线已保存为 'roc_curves.png'")

# 超参数调优示例（使用最佳模型）
if hasattr(best_model, 'get_params'):
    print(f"\n对 {best_model_name} 进行超参数调优...")

    # 根据模型类型设置不同的参数网格
    if 'Forest' in best_model_name:
        param_grid = {
            'n_estimators': [50, 100, 200],
            'max_depth': [None, 10, 20],
            'min_samples_split': [2, 5, 10]
        }
    elif 'Boosting' in best_model_name:
        param_grid = {
            'n_estimators': [50, 100, 200],
            'learning_rate': [0.01, 0.1, 0.2],
            'max_depth': [3, 5, 7]
        }
    elif best_model_name == 'SVC':
        param_grid = {
            'C': [0.1, 1, 10],
            'gamma': ['scale', 'auto'],
            'kernel': ['rbf', 'linear']
        }
    else:
        param_grid = {}  # 对于其他模型，不进行调优

    if param_grid:
        grid_search = GridSearchCV(best_model, param_grid, cv=5, scoring='accuracy', n_jobs=-1)
        grid_search.fit(X_train, y_train)

        print(f"最佳参数: {grid_search.best_params_}")
        print(f"最佳交叉验证准确率: {grid_search.best_score_:.4f}")

# 使用最佳参数重新训练模型
        best_model_tuned = grid_search.best_estimator_
        y_pred_tuned = best_model_tuned.predict(X_test)
        accuracy_tuned = accuracy_score(y_test, y_pred_tuned)
        print(f"调优后测试准确率: {accuracy_tuned:.4f}")

        # 比较调优前后的性能
        print(f"性能提升: {accuracy_tuned - results_df.loc[best_model_idx, 'Accuracy']:.4f}")

print("\n分析完成!")


#多模型比较条形图及ROC输出

import warnings
warnings.filterwarnings('ignore')  # 忽略警告

import numpy as np
import pandas as pd
import matplotlib.pyplot as plt
import seaborn as sns
from sklearn.datasets import make_classification
from sklearn.model_selection import train_test_split, GridSearchCV
from sklearn.metrics import accuracy_score, classification_report, confusion_matrix, roc_curve, auc
from sklearn.preprocessing import StandardScaler, label_binarize

# 导入其他机器学习模型
from sklearn.ensemble import RandomForestClassifier, GradientBoostingClassifier, AdaBoostClassifier, ExtraTreesClassifier
from sklearn.linear_model import LogisticRegression
from sklearn.svm import SVC
from sklearn.neighbors import KNeighborsClassifier
from sklearn.tree import DecisionTreeClassifier
from sklearn.naive_bayes import GaussianNB
from sklearn.discriminant_analysis import LinearDiscriminantAnalysis, QuadraticDiscriminantAnalysis

# 设置随机种子
np.random.seed(42)

# 创建模拟数据
print("创建模拟数据...")
X, y = make_classification(n_samples=1000, n_features=20, n_informative=10,
                           n_redundant=5, n_classes=2, random_state=42)

# 数据标准化
scaler = StandardScaler()
X_scaled = scaler.fit_transform(X)

# 分割数据
X_train, X_test, y_train, y_test = train_test_split(X_scaled, y, test_size=0.3, random_state=42)

# 初始化模型字典
models = {
    'Random Forest': RandomForestClassifier(n_estimators=100, random_state=42),
    'LightGBM': GradientBoostingClassifier(n_estimators=100, random_state=42),
    'XGBoost': AdaBoostClassifier(n_estimators=100, random_state=42),
    'Logistic Regression': LogisticRegression(max_iter=1000, random_state=42),
    'SVC': SVC(probability=True, random_state=42),
    'K-Nearest Neighbors': KNeighborsClassifier(),
    'Decision Tree': DecisionTreeClassifier(random_state=42),
}

# 训练和评估模型
results = []
print("\n训练和评估模型...")
for name, model in models.items():
    print(f"训练 {name}...")
    model.fit(X_train, y_train)
    y_pred = model.predict(X_test)
    y_pred_proba = model.predict_proba(X_test)[:, 1] if hasattr(model, "predict_proba") else None

    accuracy = accuracy_score(y_test, y_pred)

    results.append({
        'Model': name,
        'Accuracy': accuracy,
        'Model_Obj': model,
        'Y_Pred': y_pred,
        'Y_Pred_Proba': y_pred_proba
    })

    print(f"  {name} 准确率: {accuracy:.4f}")

# 创建结果数据框
results_df = pd.DataFrame(results)
print("\n模型性能比较:")
print(results_df[['Model', 'Accuracy']].sort_values('Accuracy', ascending=False))

# 可视化结果
plt.figure(figsize=(14, 8))
bars = plt.bar(results_df['Model'], results_df['Accuracy'])
plt.title('模型准确率比较', fontsize=16)
plt.xlabel('模型', fontsize=12)
plt.ylabel('准确率', fontsize=12)
plt.xticks(rotation=45, ha='right')
plt.ylim(0, 1)
plt.grid(axis='y', linestyle='--', alpha=0.7)

# 在柱状图上添加数值标签
for bar in bars:
    height = bar.get_height()
    plt.text(bar.get_x() + bar.get_width()/2., height + 0.01,
             f'{height:.3f}', ha='center', va='bottom')

plt.tight_layout()
plt.savefig('model_comparison.png', dpi=300, bbox_inches='tight')
print("\n可视化结果已保存为 'model_comparison.png'")
plt.show()

# 显示最佳模型的详细报告
best_model_idx = results_df['Accuracy'].idxmax()
best_model_name = results_df.loc[best_model_idx, 'Model']
best_model = results_df.loc[best_model_idx, 'Model_Obj']
y_pred = results_df.loc[best_model_idx, 'Y_Pred']

print(f"\n最佳模型: {best_model_name}")
print("分类报告:")
print(classification_report(y_test, y_pred))

print("混淆矩阵:")
cm = confusion_matrix(y_test, y_pred)
cm_df = pd.DataFrame(cm,
                     index=['True Negative', 'True Positive'],
                     columns=['Predicted Negative', 'Predicted Positive'])
print(cm_df)

# 特征重要性（如果可用）
if hasattr(best_model, 'feature_importances_'):
    print(f"\n{best_model_name} 特征重要性 (前10个):")
    feature_importance = pd.DataFrame({
        'feature': range(X.shape[1]),
        'importance': best_model.feature_importances_
    }).sort_values('importance', ascending=False).head(10)
    print(feature_importance)

# ROC 曲线（如果模型支持概率预测）
plt.figure(figsize=(10, 8))
for i, result in enumerate(results):
    if result['Y_Pred_Proba'] is not None:
        fpr, tpr, _ = roc_curve(y_test, result['Y_Pred_Proba'])
        roc_auc = auc(fpr, tpr)
        plt.plot(fpr, tpr, label=f'{result["Model"]} (AUC = {roc_auc:.2f})')

plt.plot([0, 1], [0, 1], 'k--')
plt.xlim([0.0, 1.0])
plt.ylim([0.0, 1.05])
plt.xlabel('False Positive Rate')
plt.ylabel('True Positive Rate')
plt.title('ROC 曲线比较')
plt.legend(loc="lower right")
plt.grid(True, alpha=0.3)
plt.savefig('roc_curves.png', dpi=300, bbox_inches='tight')
plt.show()
print("ROC 曲线已保存为 'roc_curves.png'")

# 超参数调优示例（使用最佳模型）
if hasattr(best_model, 'get_params'):
    print(f"\n对 {best_model_name} 进行超参数调优...")

    # 根据模型类型设置不同的参数网格
    if 'Forest' in best_model_name:
        param_grid = {
            'n_estimators': [50, 100, 200],
            'max_depth': [None, 10, 20],
            'min_samples_split': [2, 5, 10]
        }
    elif 'Boosting' in best_model_name:
        param_grid = {
            'n_estimators': [50, 100, 200],
            'learning_rate': [0.01, 0.1, 0.2],
            'max_depth': [3, 5, 7]
        }
    elif best_model_name == 'SVC':
        param_grid = {
            'C': [0.1, 1, 10],
            'gamma': ['scale', 'auto'],
            'kernel': ['rbf', 'linear']
        }
    else:
        param_grid = {}  # 对于其他模型，不进行调优

    if param_grid:
        grid_search = GridSearchCV(best_model, param_grid, cv=5, scoring='accuracy', n_jobs=-1)
        grid_search.fit(X_train, y_train)

        print(f"最佳参数: {grid_search.best_params_}")
        print(f"最佳交叉验证准确率: {grid_search.best_score_:.4f}")

        # 使用最佳参数重新训练模型
        best_model_tuned = grid_search.best_estimator_
        y_pred_tuned = best_model_tuned.predict(X_test)
        accuracy_tuned = accuracy_score(y_test, y_pred_tuned)
        print(f"调优后测试准确率: {accuracy_tuned:.4f}")

        # 比较调优前后的性能
        print(f"性能提升: {accuracy_tuned - results_df.loc[best_model_idx, 'Accuracy']:.4f}")

print("\n分析完成!")
```

```
#模型可解释性分析
将SHAP值矩阵传递给条形图函数可以创建一个全局特征重要性图，其中每个特征的全局重要性是其绝对平均值，涵盖所有给定样本。
#全局条形图
#lightGBM模型

import lightgbm as lgb
import numpy as np
import shap

# 假设你已经有了一个训练好的LightGBM模型
# gbm = lgb.train(...)

# 假设 'data.csv' 是你的数据文件
data = pd.read_excel('/Users/lotus/desktop/2024.07.31-FJES数据GPT用-2补充缺失值-DR.xlsx', sheet_name='Sheet1')

# 特征和目标变量
X = data[['BMI', 'HEARTRATE', 'SBP', 'DBP', 'PPG', 'MAP', 'ODIOP', 'OSIOP', 'ODNVA', 'OSNVA', 'ODBCVA0', 'OSBCVA0', 'ODS', 'OSS', 'ODC', 'OSC', 'ODSE', 'OSSE', 'HBPornot', 'DMornot', 'HGornot', 'education', 'income', 'occupation', 'outdoortimegroup', 'wearprotection', 'bloodtype', 'eyediscomfort', 'eyepain', 'darkphoneornot', 'phoneornot', 'phonetimegroup4', 'smokeornot', 'drinkornot', 'teaornot', 'whichtea', 'URBAN', 'COASTAL', 'GENDER', 'AGE', 'ODcataract', 'OScataract', 'ODmyopia', 'cataractsurgery', 'PosteriorGroup', 'OSmyopia', 'ODHM', 'OSHM', 'HEIGHT', 'WEIGHT', 'HM']]
y = data['DR']

# 划分训练集和验证集
X_train, X_val, y_train, y_val = train_test_split(X, y, test_size=0.2, random_state=42)

# 创建LightGBM数据结构
train_data = lgb.Dataset(X_train, label=y_train)
val_data = lgb.Dataset(X_val, label=y_val, reference=train_data)

# 设置训练参数
params = {
    'boosting_type': 'gbdt',
    'objective': 'regression',
    'metric': 'rmse',
    'num_leaves': 31,
    'learning_rate': 0.05,
    'feature_fraction': 0.9,
    'bagging_fraction': 0.8,
    'bagging_freq': 5,
    'verbose': -1
}

# 定义回调函数来记录验证集的性能指标
evals_result = {}

# 训练模型
gbm = lgb.train(params,
                train_data,
                num_boost_round=100,
                valid_sets=[train_data, val_data],
                valid_names=['train', 'valid'],
                callbacks=[lgb.record_evaluation(evals_result)])


# 创建SHAP解释器
explainer = shap.TreeExplainer(gbm)

# 计算SHAP值
shap_values = explainer.shap_values(X)

# 输出SHAP值
print(shap_values)
plt.show()
# 创建全局特征重要性图
shap.summary_plot(shap_values, X_test, plot_type="bar")
```

```
SHAP值计算

import matplotlib.pyplot as plt
import numpy as np

# Data extracted from the image
features = [
    "DMonot", "Age", "PPG", "OSNVA", "HEIGHT", "HEARTRATE", "OSBCVA0", "ODNVA",
    "MAP", "cataractsurgery", "DBP", "education", "OScataract", "ODBCVA0",
    "ODSE", "BMI", "WEIGHT", "ODS", "OSC", "tearnot"
]
shap_values = [
    0.080, 0.035, 0.030, 0.027, 0.024, 0.022, 0.018, 0.016, 0.015, 0.014, 0.013,
    0.012, 0.011, 0.010, 0.009, 0.008, 0.007, 0.006, 0.005, 0.004
]

# Sorting the data
sorted_indices = np.argsort(shap_values)
sorted_features = [features[i] for i in sorted_indices]
sorted_shap_values = [shap_values[i] for i in sorted_indices]

# Creating a gradient color map
colors = plt.cm.viridis(np.linspace(0, 1, len(shap_values)))

# Plotting the sorted data
plt.figure(figsize=(8, 6))
plt.barh(sorted_features, sorted_shap_values, color=colors)
plt.xlabel("mean(|SHAP value|) (average impact on model output magnitude)")
plt.title("Feature Importance (Sorted)")
plt.show()
```

#ROC

#lightgbm模型

```
from lightgbm import LGBMClassifier
from sklearn.metrics import RocCurveDisplay
import matplotlib.pyplot as plt

# Assuming X_train, y_train, X_test, and y_test are your data
lgb_model = LGBMClassifier()
lgb_model.fit(X_train, y_train)

# Get the predicted probabilities for each class
y_score = lgb_model.predict_proba(X_test)

# Number of classes
n_classes = len(lgb_model.classes_)

# Create a figure to hold all the ROC curves
plt.figure(figsize=(10, 8))

# Plot ROC curve for each class
for i, class_label in enumerate(lgb_model.classes_):
    # Convert labels to binary for the current class
    y_true_binary = (y_test == class_label).astype(int)

    # Extract the scores for the current class
    y_score_binary = y_score[:, i]

    # Create a ROC curve display
    RocCurveDisplay.from_predictions(y_true_binary, y_score_binary)

plt.plot([0, 1], [0, 1], 'k--')  # Plot the diagonal line
plt.show()
```

#模型性能比较

import numpy as np

import pandas as pd

import matplotlib.pyplot as plt

import seaborn as sns

from sklearn.metrics import accuracy_score, precision_score, recall_score, f1_score

from sklearn.metrics import classification_report, confusion_matrix

from sklearn.datasets import make_classification

\# 生成模拟数据用于演示

np.random.seed(42)

X, y = make_classification(n_samples=1000, n_features=20, n_informative=15,

n_redundant=5, n_classes=3, random_state=42)

\# 分割训练测试集

from sklearn.model_selection import train_test_split

X_train, X_test, y_train, y_test = train_test_split(X, y, test_size=0.3, random_state=42)

\# 训练几个模型用于演示

from sklearn.ensemble import RandomForestClassifier

from sklearn.linear_model import LogisticRegression

from sklearn.svm import SVC

from sklearn.neighbors import KNeighborsClassifier

from sklearn.tree import DecisionTreeClassifier

\# 初始化模型

models = {

'Random Forest': RandomForestClassifier(random_state=42),

'Logistic Regression': LogisticRegression(random_state=42, max_iter=1000),

'SVC': SVC(random_state=42),

'KNN': KNeighborsClassifier(),

'Decision Tree': DecisionTreeClassifier(random_state=42)

}

\# 训练模型并获取预测

predictions = {}

for name, model in models.items():

model.fit(X_train, y_train)

predictions[name] = model.predict(X_test)

def evaluate_multiclass_performance(y_true, y_pred, model_name=""):

"""

评估多分类模型的性能指标

参数:

y_true: 真实标签

y_pred: 预测标签

model_name: 模型名称（可选）

返回:

results_dict: 包含所有评估指标的字典

"""

\# 计算总体准确度

accuracy = accuracy_score(y_true, y_pred)

\# 计算每个类别的精确度、召回率和F1分数

precision_per_class = precision_score(y_true, y_pred, average=None, zero_division=0)

recall_per_class = recall_score(y_true, y_pred, average=None, zero_division=0)

f1_per_class = f1_score(y_true, y_pred, average=None, zero_division=0)

\# 计算宏观平均指标（每个类别的平等权重）

macro_precision = precision_score(y_true, y_pred, average='macro', zero_division=0)

macro_recall = recall_score(y_true, y_pred, average='macro', zero_division=0)

macro_f1 = f1_score(y_true, y_pred, average='macro', zero_division=0)

\# 计算微观平均指标（每个样本的平等权重）

micro_precision = precision_score(y_true, y_pred, average='micro', zero_division=0)

micro_recall = recall_score(y_true, y_pred, average='micro', zero_division=0)

micro_f1 = f1_score(y_true, y_pred, average='micro', zero_division=0)

\# 计算加权平均指标（按每个类别的样本数加权）

weighted_precision = precision_score(y_true, y_pred, average='weighted', zero_division=0)

weighted_recall = recall_score(y_true, y_pred, average='weighted', zero_division=0)

weighted_f1 = f1_score(y_true, y_pred, average='weighted', zero_division=0)

\# 创建结果字典

results_dict = {

'model': model_name,

'accuracy': accuracy,

'precision_per_class': precision_per_class,

'recall_per_class': recall_per_class,

'f1_per_class': f1_per_class,

'macro_precision': macro_precision,

'macro_recall': macro_recall,

'macro_f1': macro_f1,

'micro_precision': micro_precision,

'micro_recall': micro_recall,

'micro_f1': micro_f1,

'weighted_precision': weighted_precision,

'weighted_recall': weighted_recall,

'weighted_f1': weighted_f1

}

return results_dict

def print_detailed_report(y_true, y_pred, model_name="", class_names=None):

"""

打印详细的分类报告和混淆矩阵

参数:

y_true: 真实标签

y_pred: 预测标签

model_name: 模型名称（可选）

class_names: 类别名称列表（可选）

"""

if model_name:

print(f"===== {model_name} 性能报告 =====")

\# 打印分类报告

print("\n分类报告:")

print(classification_report(y_true, y_pred, target_names=class_names, zero_division=0))

\# 打印混淆矩阵

print("混淆矩阵:")

cm = confusion_matrix(y_true, y_pred)

cm_df = pd.DataFrame(cm)

if class_names:

cm_df.columns = class_names

cm_df.index = class_names

print(cm_df)

\# 计算并打印额外指标

results = evaluate_multiclass_performance(y_true, y_pred, model_name)

print(f"\n总体准确度: {results['accuracy']:.4f}")

print(f"宏观平均F1分数: {results['macro_f1']:.4f}")

print(f"微观平均F1分数: {results['micro_f1']:.4f}")

print(f"加权平均F1分数: {results['weighted_f1']:.4f}")

return results

\# 存储所有模型的结果

all_results = []

\# 为每个模型计算性能指标

for model_name, y_pred in predictions.items():

results = print_detailed_report(y_test, y_pred, model_name, class_names=['Class 0', 'Class 1', 'Class 2'])

all_results.append(results)

print("\n" + "="*50 + "\n")

\# 创建比较表格

results_df = pd.DataFrame(all_results)

comparison_table = results_df[['model', 'accuracy', 'macro_precision', 'macro_recall', 'macro_f1',

'micro_precision', 'micro_recall', 'micro_f1']]

print("\n===== 模型性能比较 =====")

print(comparison_table.round(4))

\# 可视化比较结果

plt.figure(figsize=(14, 8))

\# 创建子图

plt.subplot(2, 2, 1)

plt.bar(comparison_table['model'], comparison_table['accuracy'])

plt.title('模型准确度比较')

plt.xticks(rotation=45)

plt.grid(True, alpha=0.3)

plt.subplot(2, 2, 2)

plt.bar(comparison_table['model'], comparison_table['macro_f1'])

plt.title('宏观平均F1分数比较')

plt.xticks(rotation=45)

plt.grid(True, alpha=0.3)

plt.subplot(2, 2, 3)

plt.bar(comparison_table['model'], comparison_table['micro_f1'])

plt.title('微观平均F1分数比较')

plt.xticks(rotation=45)

plt.grid(True, alpha=0.3)

plt.subplot(2, 2, 4)

x = np.arange(len(comparison_table))

width = 0.25

plt.bar(x - width, comparison_table['accuracy'], width, label='准确度')

plt.bar(x, comparison_table['macro_f1'], width, label='宏观F1')

plt.bar(x + width, comparison_table['micro_f1'], width, label='微观F1')

plt.title('模型性能综合比较')

plt.xticks(x, comparison_table['model'], rotation=45)

plt.legend()

plt.grid(True, alpha=0.3)

plt.tight_layout()

plt.show()

\# 保存结果到CSV文件

comparison_table.to_csv('model_performance_comparison.csv', index=False)

print("模型性能比较结果已保存到 'model_performance_comparison.csv'")

总结一下：1.数据准备；2.数据探索性分析；3.数据清洗；4.模型构建与评估：机器学习的临床预测模型构建过程！！！

#运行unsupervised feature grouping (FA)的代码

```
import numpy as np
import matplotlib.pyplot as plt
from sklearn.decomposition import FactorAnalysis
from sklearn.preprocessing import StandardScaler
import pandas as pd

# 假设您已经有一个名为X的DataFrame
# 这里我创建示例数据以便代码可运行
np.random.seed(42)
data_size = 8211
feature_names = ['BMI', 'HEARTRATE', 'SBP', 'DBP', 'PPG', 'MAP', 'ODIOP', 'OSIOP', 'ODNVA', 'OSNVA',
                 'ODBCVA0', 'OSBCVA0', 'ODS', 'OSS', 'ODC', 'OSC', 'ODSE', 'OSSE', 'HBPornot', 'DMornot',
                 'HGornot', 'education', 'income', 'occupation', 'outdoortimegroup', 'wearprotection',
                 'bloodtype', 'eyediscomfort', 'eyepain', 'darkphoneornot', 'phoneornot', 'phonetimegroup4',
                 'smokeornot', 'drinkornot', 'teaornot', 'whichtea', 'URBAN', 'COASTAL', 'GENDER', 'Age',
                 'ODcataract', 'OScataract', 'ODmyopia', 'cataractsurgery', 'PosteriorGroup', 'OSmyopia',
                 'ODHM', 'OSHM', 'HEIGHT', 'WEIGHT', 'HM']

# 创建模拟数据 (在实际应用中替换为您的真实数据)
X = pd.DataFrame(np.random.randn(data_size, len(feature_names)),
                 columns=feature_names)

# 1. 数据标准化
scaler = StandardScaler()
X_scaled = scaler.fit_transform(X)

# 2. 执行因子分析
n_components = 2
fa = FactorAnalysis(n_components=n_components,
                    rotation='varimax',  # 添加旋转使因子更易解释
                    max_iter=1000,
                    random_state=42)
X_transformed = fa.fit_transform(X_scaled)

# 3. 输出结果
print("原始特征维度:", X.shape)
print("转换后维度:", X_transformed.shape)
print("\n因子载荷矩阵 (特征 x 因子):")
loadings = pd.DataFrame(fa.components_.T,  # 转置后: 行=特征, 列=因子
                        index=feature_names,
                        columns=[f'Factor {i+1}' for i in range(n_components)])
print(loadings)

# 4. 可视化因子分组结果
plt.figure(figsize=(12, 8))
plt.scatter(X_transformed[:, 0], X_transformed[:, 1], alpha=0.5)
plt.xlabel('Factor 1')
plt.ylabel('Factor 2')
plt.title('Factor Analysis Results')
plt.grid(True)
plt.show()

# 5. 解释因子含义
print("\n因子解释:")
for i in range(n_components):
    print(f"\n因子 {i+1} 主要受以下特征影响:")
    # 获取绝对值最大的5个特征
    component = fa.components_[i]
    top_features_idx = np.argsort(np.abs(component))[::-1][:5]

    for idx in top_features_idx:
        feature = feature_names[idx]
        weight = component[idx]
        print(f"- {feature} (权重: {weight:.3f})")

# 6. 可视化因子载荷矩阵
plt.figure(figsize=(14, 10))
plt.imshow(np.abs(fa.components_), cmap='viridis', aspect='auto')
plt.colorbar(label='因子载荷绝对值')
plt.xticks(range(n_components), [f'Factor {i+1}' for i in range(n_components)])
plt.yticks(range(len(feature_names)), feature_names, fontsize=8)
plt.title('因子载荷矩阵热力图')
plt.tight_layout()
plt.show()

# 7. 保存结果到CSV (修复了类型错误)
# 创建一个新的DataFrame来保存结果，避免修改原始loadings
results_df = loadings.copy()

# 计算每个特征归属的主要因子及其载荷值
abs_loadings = results_df.abs()
results_df['Max Factor'] = abs_loadings.idxmax(axis=1)
results_df['Loading Value'] = abs_loadings.max(axis=1)

# 按最大载荷值排序
results_df = results_df.sort_values(by='Loading Value', ascending=False)

results_df.to_csv('factor_analysis_results.csv')
print("\n因子分析结果已保存到 'factor_analysis_results.csv'")

# 8. 显示最重要的特征
print("\n最重要的特征（按因子载荷绝对值排序）:")
print(results_df[['Max Factor', 'Loading Value']].head(10))

# 9. 因子相关性分析
print("\n因子相关性分析:")
factor_corr = pd.DataFrame(X_transformed, columns=[f'Factor {i+1}' for i in range(n_components)])
print(factor_corr.corr())


原始特征维度: (8211, 51)
转换后维度: (8211, 2)
因子载荷矩阵 (特征 x 因子):
                  Factor 1  Factor 2
BMI              -0.009499  0.042437
HEARTRATE        -0.001314 -0.004954
SBP               0.012809 -0.033540
DBP               0.006260 -0.050092
PPG              -0.069446 -0.029294
MAP               0.017475 -0.004502
ODIOP            -0.054758 -0.023578
OSIOP            -0.015496  0.025382
ODNVA            -0.075867  0.008932
OSNVA             0.060520 -0.008818
ODBCVA0           0.032934 -0.045849
OSBCVA0           0.005732  0.041402
ODS               0.059567 -0.032376
OSS              -0.028622 -0.029410
ODC               0.076602  0.024887
OSC               0.034228  0.013601
ODSE              0.027435  0.009929
OSSE              0.019717 -0.107258
HBPornot          0.038817  0.060246
DMornot          -0.018168 -0.057679
HGornot          -0.004687  0.070973
education        -0.021159  0.031203
income           -0.048317  0.018916
occupation       -0.014606 -0.070992
outdoortimegroup -0.032635  0.022783
wearprotection   -0.007202 -0.029847
bloodtype         0.074731  0.002908
eyediscomfort    -0.020651 -0.007274
eyepain          -0.008341  0.007286
darkphoneornot   -0.031911 -0.020325
phoneornot       -0.041662  0.065442
phonetimegroup4   0.024914  0.013839
smokeornot        0.058583 -0.011100
drinkornot        0.041191 -0.051872
teaornot          0.044062  0.003456
whichtea          0.017819  0.011970
URBAN            -0.062935 -0.003818
COASTAL           0.027289 -0.033426
GENDER            0.033003 -0.013580
Age               0.035377 -0.040799
ODcataract        0.066590  0.017023
OScataract        0.014708  0.039970
ODmyopia          0.096566 -0.027991
cataractsurgery   0.026548  0.047254
PosteriorGroup    0.000378 -0.013899
OSmyopia         -0.073467 -0.086661
ODHM             -0.019560 -0.070018
OSHM             -0.019323 -0.029653
HEIGHT           -0.009972  0.068335
WEIGHT           -0.061697 -0.024662
HM               -0.060041  0.006483
```

因子解释:

因子 1 主要受以下特征影响:

- ODmyopia (权重: 0.097)

- ODC (权重: 0.077)

- ODNVA (权重: -0.076)

- bloodtype (权重: 0.075)

- OSmyopia (权重: -0.073)

因子 2 主要受以下特征影响:

- OSSE (权重: -0.107)

- OSmyopia (权重: -0.087)

- occupation (权重: -0.071)

- HGornot (权重: 0.071)

- ODHM (权重: -0.070)

```
因子分析结果已保存到 'factor_analysis_results.csv'
最重要的特征（按因子载荷绝对值排序）:
           Max Factor  Loading Value
OSSE         Factor 2       0.107258
ODmyopia     Factor 1       0.096566
OSmyopia     Factor 2       0.086661
ODC          Factor 1       0.076602
ODNVA        Factor 1       0.075867
bloodtype    Factor 1       0.074731
occupation   Factor 2       0.070992
HGornot      Factor 2       0.070973
ODHM         Factor 2       0.070018
PPG          Factor 1       0.069446
因子相关性分析:
          Factor 1  Factor 2
Factor 1  1.000000  0.035244
Factor 2  0.035244  1.000000
```

#运行highly variable feature selection (HVFS)的代码

```
import numpy as np
import pandas as pd
import matplotlib.pyplot as plt
from sklearn.preprocessing import StandardScaler
from sklearn.feature_selection import VarianceThreshold

# 定义您的特征名称列表
feature_names = [
    'BMI', 'HEARTRATE', 'SBP', 'DBP', 'PPG', 'MAP', 'ODIOP', 'OSIOP', 'ODNVA', 'OSNVA',
    'ODBCVA0', 'OSBCVA0', 'ODS', 'OSS', 'ODC', 'OSC', 'ODSE', 'OSSE', 'HBPornot', 'DMornot',
    'HGornot', 'education', 'income', 'occupation', 'outdoortimegroup', 'wearprotection',
    'bloodtype', 'eyediscomfort', 'eyepain', 'darkphoneornot', 'phoneornot', 'phonetimegroup4',
    'smokeornot', 'drinkornot', 'teaornot', 'whichtea', 'URBAN', 'COASTAL', 'GENDER', 'AGE',
    'ODcataract', 'OScataract', 'ODmyopia', 'cataractsurgery', 'PosteriorGroup', 'OSmyopia',
    'ODHM', 'OSHM', 'HEIGHT', 'WEIGHT', 'HM'
]

def highly_variable_feature_selection(X, feature_names, n_top_features=10, plot=True):
    """
    高度可变特征选择 (HVFS)

    参数:
    X : DataFrame 或 array-like, 形状 (n_samples, n_features)
        输入数据矩阵
    feature_names : list
        特征名称列表
    n_top_features : int, 可选 (默认=10)
        选择的最具可变性的特征数量
    plot : bool, 可选 (默认=True)
        是否绘制特征可变性可视化图

    返回:
    selected_features : list
        选择的高度可变特征名称
    variance_df : DataFrame
        包含特征方差信息的DataFrame
    """
    # 确保输入是DataFrame
    if not isinstance(X, pd.DataFrame):
        X = pd.DataFrame(X, columns=feature_names)

    # 1. 数据标准化 (可选，取决于数据特性)
    scaler = StandardScaler()
    X_scaled = scaler.fit_transform(X)
    X_scaled = pd.DataFrame(X_scaled, columns=feature_names)

    # 2. 计算每个特征的方差
    variances = np.var(X_scaled, axis=0)

    # 3. 创建方差信息DataFrame
    variance_df = pd.DataFrame({
        'Feature': feature_names,
        'Variance': variances
    }).sort_values('Variance', ascending=False)

    # 4. 选择高度可变特征
    selected_features = variance_df['Feature'].head(n_top_features).tolist()

    # 5. 可视化
    if plot:
        plt.figure(figsize=(14, 10))

        # 方差分布图
        plt.subplot(2, 1, 1)
        # 只显示前20个特征的名称，避免拥挤
        top_features = variance_df.head(20)
        plt.bar(top_features['Feature'], top_features['Variance'], color='skyblue')
        plt.axhline(y=np.median(variances), color='r', linestyle='--',
                    label=f'Median Variance: {np.median(variances):.2f}')
        plt.title('Top 20 Features by Variance')
        plt.xlabel('Features')
        plt.ylabel('Variance')
        plt.xticks(rotation=45, ha='right')
        plt.legend()

        # 方差排序图
        plt.subplot(2, 1, 2)
        sorted_variances = np.sort(variances)[::-1]
        plt.plot(sorted_variances, 'o-', color='darkblue')
        plt.axvline(x=n_top_features-1, color='g', linestyle='--',
                    label=f'Top {n_top_features} Features')
        plt.title('Sorted Feature Variances')
        plt.xlabel('Feature Rank')
        plt.ylabel('Variance')
        plt.legend()
        plt.tight_layout()
        plt.savefig('highly_variable_features.png', dpi=300)
        plt.show()

        # 打印选择结果
        print(f"\nTop {n_top_features} highly variable features:")
        print(variance_df.head(n_top_features))

    return selected_features, variance_df

# =====================
# 使用示例 - 使用您的真实数据
# =====================

# 1. 创建模拟数据 (在实际应用中替换为您的真实数据)
np.random.seed(42)
data_size = 8211  # 与您的实际样本量一致
n_features = len(feature_names)

# 创建不同方差水平的特征
X = np.zeros((data_size, n_features))
for i in range(n_features):
    # 为不同特征设置不同的方差水平
    variance_level = np.random.uniform(0.1, 10.0)
    X[:, i] = np.random.normal(0, variance_level, data_size)

# 创建DataFrame
X = pd.DataFrame(X, columns=feature_names)

# 2. 执行高度可变特征选择
selected_features, variance_df = highly_variable_feature_selection(
    X,
    feature_names,
    n_top_features=15,  # 选择前15个高度可变特征
    plot=True
)

# 3. 使用sklearn的VarianceThreshold进行特征选择
# 设置方差阈值 (例如，保留方差大于1.0的特征)
thresholder = VarianceThreshold(threshold=1.0)
X_high_variance = thresholder.fit_transform(X)

# 获取被保留的特征索引
retained_indices = thresholder.get_support(indices=True)
retained_features = [feature_names[i] for i in retained_indices]

print(f"\nFeatures retained with variance threshold (threshold=1.0): {len(retained_features)}")
print(retained_features)

# 4. 保存结果
variance_df.to_csv('feature_variances.csv', index=False)
print("\n特征方差结果已保存到 'feature_variances.csv'")

# 5. 分析结果
print("\n分析报告:")
print(f"- 总特征数: {n_features}")
print(f"- 选择的高度可变特征数: {len(selected_features)}")
print(f"- 基于阈值保留的特征数: {len(retained_features)}")
print(f"- 最高方差特征: {variance_df.iloc[0]['Feature']} (方差={variance_df.iloc[0]['Variance']:.4f})")
print(f"- 最低方差特征: {variance_df.iloc[-1]['Feature']} (方差={variance_df.iloc[-1]['Variance']:.4f})")

# 6. 实际数据应用提示
print("\n" + "="*80)
print("当使用您的真实数据时：")
print("1. 替换模拟数据部分为您的真实数据集")
print("2. 确保数据矩阵X的列数与feature_names的长度一致")
print("3. 如果数据有缺失值，请先处理缺失值")
print("="*80)
```

```
Top 15 highly variable features:
               Feature  Variance
URBAN            URBAN       1.0
education    education       1.0
OSNVA            OSNVA       1.0
OSSE              OSSE       1.0
HM                  HM       1.0
DBP                DBP       1.0
OSHM              OSHM       1.0
OSS                OSS       1.0
HBPornot      HBPornot       1.0
drinkornot  drinkornot       1.0
AGE                AGE       1.0
HEARTRATE    HEARTRATE       1.0
eyepain        eyepain       1.0
ODNVA            ODNVA       1.0
ODHM              ODHM       1.0

Features retained with variance threshold (threshold=1.0): 48
['BMI', 'HEARTRATE', 'SBP', 'DBP', 'PPG', 'MAP', 'ODIOP', 'OSIOP', 'ODNVA', 'OSNVA', 'ODBCVA0', 'OSBCVA0', 'ODS', 'OSS', 'ODC', 'OSC', 'ODSE', 'OSSE', 'HBPornot', 'DMornot', 'HGornot', 'education', 'income', 'occupation', 'outdoortimegroup', 'wearprotection', 'bloodtype', 'eyepain', 'darkphoneornot', 'phoneornot', 'phonetimegroup4', 'smokeornot', 'teaornot', 'whichtea', 'URBAN', 'COASTAL', 'GENDER', 'AGE', 'ODcataract', 'OScataract', 'cataractsurgery', 'PosteriorGroup', 'OSmyopia', 'ODHM', 'OSHM', 'HEIGHT', 'WEIGHT', 'HM']

特征方差结果已保存到 'feature_variances.csv'

分析报告:
- 总特征数: 51
- 选择的高度可变特征数: 15
- 基于阈值保留的特征数: 48
- 最高方差特征: URBAN (方差=1.0000)
- 最低方差特征: BMI (方差=1.0000)

================================================================================
当使用您的真实数据时：
1. 替换模拟数据部分为您的真实数据集
2. 确保数据矩阵X的列数与feature_names的长度一致
3. 如果数据有缺失值，请先处理缺失值
================================================================================


# 在机器学习流程中使用HVFS
from sklearn.pipeline import Pipeline
from sklearn.ensemble import RandomForestClassifier

# 创建包含HVFS的管道
pipeline = Pipeline([
    ('variance_threshold', VarianceThreshold(threshold=0.5)),
    ('classifier', RandomForestClassifier())
])
```

****#特征相关性分析****

```
# 正确的列选择方式：使用列名的列表
selected_columns = [
    'BMI', 'HEARTRATE', 'SBP', 'DBP', 'PPG', 'MAP', 'ODIOP', 'OSIOP', 'ODNVA', 'OSNVA',
    'ODBCVA0', 'OSBCVA0', 'ODS', 'OSS', 'ODC', 'OSC', 'ODSE', 'OSSE', 'HBPornot', 'DMornot',
    'HGornot', 'education', 'income', 'occupation', 'outdoortimegroup', 'wearprotection',
    'bloodtype', 'eyepain', 'darkphoneornot', 'phoneornot', 'phonetimegroup4',
    'smokeornot', 'teaornot', 'whichtea', 'URBAN', 'COASTAL', 'GENDER', 'AGE',
    'ODcataract', 'OScataract', 'cataractsurgery', 'PosteriorGroup', 'OSmyopia',
    'ODHM', 'OSHM', 'HEIGHT', 'WEIGHT', 'HM'
]

# 确保所有列都存在于DataFrame中
missing_columns = [col for col in selected_columns if col not in X.columns]
if missing_columns:
    print(f"警告: 以下列在DataFrame中不存在: {missing_columns}")
    selected_columns = [col for col in selected_columns if col in X.columns]

# 计算相关系数矩阵
correlation_matrix = X[selected_columns].corr()

# 创建热力图
plt.figure(figsize=(20, 18))  # 增加图形尺寸以适应更多特征
sns.heatmap(correlation_matrix, annot=False, cmap='coolwarm', fmt='.2f',
            cbar_kws={'shrink': 0.8}, square=True)
plt.title('Selected Features Correlation Matrix')
plt.xticks(rotation=45, ha='right')  # 旋转x轴标签
plt.yticks(rotation=0)  # 保持y轴标签水平
plt.tight_layout()
plt.savefig('correlation_matrix.png', dpi=300, bbox_inches='tight')
plt.show()
```

****#与目标变量的关系****

```
import numpy as np
import pandas as pd
from sklearn.feature_selection import mutual_info_classif
import matplotlib.pyplot as plt
import seaborn as sns

# 定义特征名称列表
feature_names = [
    'BMI', 'HEARTRATE', 'SBP', 'DBP', 'PPG', 'MAP', 'ODIOP', 'OSIOP', 'ODNVA', 'OSNVA',
    'ODBCVA0', 'OSBCVA0', 'ODS', 'OSS', 'ODC', 'OSC', 'ODSE', 'OSSE', 'HBPornot', 'DMornot',
    'HGornot', 'education', 'income', 'occupation', 'outdoortimegroup', 'wearprotection',
    'bloodtype', 'eyediscomfort', 'eyepain', 'darkphoneornot', 'phoneornot', 'phonetimegroup4',
    'smokeornot', 'drinkornot', 'teaornot', 'whichtea', 'URBAN', 'COASTAL', 'GENDER', 'AGE',
    'ODcataract', 'OScataract', 'ODmyopia', 'cataractsurgery', 'PosteriorGroup', 'OSmyopia',
    'ODHM', 'OSHM', 'HEIGHT', 'WEIGHT', 'HM'
]

# 假设您的真实数据
# X 应该是 (n_samples, n_features) 的DataFrame或数组
# y 应该是 (n_samples,) 的目标变量

# 为了演示，创建与您数据集规模一致的模拟数据
np.random.seed(42)
n_samples = 8211  # 与您的实际样本量一致
n_features = len(feature_names)

# 创建特征矩阵 (8211个样本，51个特征)
X = pd.DataFrame(np.random.randn(n_samples, n_features),
                 columns=feature_names)

# 创建目标变量 (8211个样本)
y = np.random.choice([0, 1], size=n_samples, p=[0.7, 0.3])  # 二元分类目标

# 1. 检查数据形状
print(f"X shape: {X.shape}")  # 应该是 (8211, 51)
print(f"y shape: {y.shape}")  # 应该是 (8211,)
print(f"特征数量: {len(feature_names)}")

# 2. 计算互信息
print("\n计算互信息中...")
mi_scores = mutual_info_classif(X, y, random_state=42)
mi_df = pd.DataFrame({
    'Feature': feature_names,
    'MI_Score': mi_scores
}).sort_values('MI_Score', ascending=False)

# 3. 显示结果
print("\nTop 10 features by mutual information:")
print(mi_df.head(10))

# 4. 可视化
plt.figure(figsize=(12, 8))
sns.barplot(x='MI_Score', y='Feature', data=mi_df.head(20), palette='viridis')
plt.title('Top 20 Features by Mutual Information')
plt.xlabel('Mutual Information Score')
plt.ylabel('Feature')
plt.tight_layout()
plt.savefig('mutual_information_top_features.png', dpi=300)
plt.show()

# 5. 保存结果
mi_df.to_csv('mutual_information_scores.csv', index=False)
print("\n互信息结果已保存到 'mutual_information_scores.csv'")

# 6. 阈值选择 (选择MI分数大于平均值的特征)
mean_mi = mi_df['MI_Score'].mean()
selected_features = mi_df[mi_df['MI_Score'] > mean_mi]['Feature'].tolist()
print(f"\nSelected {len(selected_features)} features with MI > mean ({mean_mi:.4f}):")
print(selected_features)

# 7. 特征分布分析 (按目标类别)
plt.figure(figsize=(14, 10))
for i, feature in enumerate(mi_df['Feature'].head(4)):
    plt.subplot(2, 2, i+1)
    sns.kdeplot(data=X, x=feature, hue=y, palette='coolwarm', fill=True, common_norm=False)
    plt.title(f'Distribution of {feature} (MI={mi_df.loc[mi_df["Feature"]==feature, "MI_Score"].values[0]:.4f})')
    plt.xlabel(feature)
    plt.ylabel('Density')
plt.tight_layout()
plt.savefig('feature_distributions_by_class.png', dpi=300)
plt.show()

# 8. 特征重要性分析
plt.figure(figsize=(10, 6))
sns.scatterplot(x=range(len(mi_df)), y=mi_df['MI_Score'], s=100, alpha=0.7)
plt.axhline(y=mean_mi, color='r', linestyle='--', label=f'Mean MI: {mean_mi:.4f}')
plt.title('Mutual Information Scores Distribution')
plt.xlabel('Feature Rank')
plt.ylabel('Mutual Information Score')
plt.legend()
plt.grid(True)
plt.tight_layout()
plt.savefig('mi_scores_distribution.png', dpi=300)
plt.show()

# 9. 实际数据应用提示
print("\n" + "="*80)
print("当使用您的真实数据时：")
print("1. 替换模拟数据部分为您的真实数据集")
print("2. 确保目标变量y是二元分类变量（0和1）")
print("3. 如果数据有缺失值，请先处理缺失值")
print("4. 分类变量需要适当编码（例如使用独热编码）")
print("="*80)


X shape: (8211, 51)
y shape: (8211,)
特征数量: 51

计算互信息中...

Top 10 features by mutual information:
             Feature  MI_Score
44    PosteriorGroup  0.011210
26         bloodtype  0.010486
24  outdoortimegroup  0.009192
4                PPG  0.008116
34          teaornot  0.007284
35          whichtea  0.006857
1          HEARTRATE  0.006040
3                DBP  0.005625
9              OSNVA  0.005264
0                BMI  0.004495
```

互信息结果已保存到 'mutual_information_scores.csv'

Selected 15 features with MI > mean (0.0020):

['PosteriorGroup', 'bloodtype', 'outdoortimegroup', 'PPG', 'teaornot', 'whichtea', 'HEARTRATE', 'DBP', 'OSNVA', 'BMI', 'AGE', 'OSS', 'education', 'phoneornot', 'OSC']

当使用您的真实数据时：

1. 替换模拟数据部分为您的真实数据集

2. 确保目标变量y是二元分类变量（0和1）

3. 如果数据有缺失值，请先处理缺失值

4. 分类变量需要适当编码（例如使用独热编码）

#适合本研究的运行nonparametric correlation methods such as Spearman’s correlation的代码

### #1. 基础Spearman相关分析

```
import pandas as pd
import numpy as np
import seaborn as sns
import matplotlib.pyplot as plt
from scipy.stats import spearmanr

# 加载数据（替换为您的数据）
# df = pd.read_csv('your_data.csv')
# 示例数据
np.random.seed(42)
df = pd.DataFrame({
    'BMI': np.random.normal(24, 3, 100),
    'SBP': np.random.normal(130, 15, 100),
    'DBP': np.random.normal(80, 10, 100),
    'ODcataract': np.random.choice([0, 1, 2], 100, p=[0.7, 0.2, 0.1]),
    'OScataract': np.random.choice([0, 1, 2], 100, p=[0.7, 0.2, 0.1]),
    'Age': np.random.randint(40, 80, 100),
    'HEIGHT': np.random.normal(170, 10, 100),
    'WEIGHT': np.random.normal(70, 15, 100),
    'HM': np.random.choice([0, 1], 100, p=[0.8, 0.2])
})

# 1. 计算所有变量之间的Spearman相关矩阵
corr_matrix = df.corr(method='spearman')

# 2. 可视化相关矩阵
plt.figure(figsize=(12, 10))
sns.heatmap(corr_matrix,
            annot=True,
            fmt=".2f",
            cmap='coolwarm',
            center=0,
            mask=np.triu(np.ones_like(corr_matrix, dtype=bool)))
plt.title('Spearman Correlation Matrix')
plt.tight_layout()
plt.show()

# 3. 计算显著性水平
p_matrix = pd.DataFrame(np.zeros_like(corr_matrix),
                        columns=corr_matrix.columns,
                        index=corr_matrix.index)

for i, col1 in enumerate(df.columns):
    for j, col2 in enumerate(df.columns):
        if i < j:  # 只计算上三角
            corr, p_value = spearmanr(df[col1], df[col2])
            p_matrix.loc[col1, col2] = p_value

# 4. 保存结果
corr_matrix.to_csv('spearman_correlation_matrix.csv')
p_matrix.to_csv('spearman_p_values.csv')

print("Spearman相关矩阵和p值已保存为CSV文件")
```

Spearman相关矩阵和p值已保存为CSV文件

### #2. 目标导向的Spearman分析（与特定结果变量相关）

```
# 假设我们关注与'BMI'（可替换为其它任一变量）的相关性
target = 'BMI'

# 1. 计算每个特征与目标变量的Spearman相关
correlations = []
p_values = []

for feature in df.columns:
    if feature != target:
        corr, p_val = spearmanr(df[feature], df[target])
        correlations.append(corr)
        p_values.append(p_val)

# 2. 创建结果DataFrame
results = pd.DataFrame({
    'Feature': [col for col in df.columns if col != target],
    'Spearman_Correlation': correlations,
    'P_Value': p_values,
    'Significance': ['*' if p < 0.05 else '' for p in p_values]
})

# 3. 按相关性绝对值排序
results = results.sort_values(by='Spearman_Correlation',
                              key=lambda x: abs(x),
                              ascending=False)

# 4. 可视化
plt.figure(figsize=(10, 8))
sns.barplot(x='Spearman_Correlation',
            y='Feature',
            data=results,
            palette='viridis')
plt.axvline(0, color='black', linestyle='--')
plt.title(f'Spearman Correlation with {target}')
plt.xlabel('Correlation Coefficient')
plt.ylabel('Features')
plt.tight_layout()
plt.show()

# 5. 保存结果
results.to_csv(f'spearman_correlation_with_{target}.csv', index=False)
print(f"\n与{target}的Spearman相关结果:")
print(results.head(10))
```

```
与BMI的Spearman相关结果:
      Feature  Spearman_Correlation   P_Value Significance
1         DBP              0.176694  0.078648
7          HM             -0.128178  0.203758
0         SBP             -0.107963  0.284987
4         Age              0.038118  0.706527
5      HEIGHT             -0.037852  0.708484
6      WEIGHT             -0.022490  0.824234
2  ODcataract              0.020106  0.842611
3  OScataract             -0.006559  0.948357
```

### #3. 高级分析：部分Spearman相关（控制混杂因素）

```
from scipy.stats import pearsonr

def partial_spearman(x, y, z):
    """
    计算控制变量z后x和y的部分Spearman相关
    """
    # 1. 计算残差
    res_x = x - spearmanr(x, z)[0] * z
    res_y = y - spearmanr(y, z)[0] * z

    # 2. 计算残差之间的Spearman相关
    return spearmanr(res_x, res_y)

# 示例：控制年龄后，分析BMI与HM的相关性
x = df['BMI']
y = df['HM']
z = df['Age']  # 控制变量

corr, p_val = partial_spearman(x, y, z)
print(f"控制年龄后，BMI与HM的部分Spearman相关: {corr:.3f} (p={p_val:.4f})")

# 批量分析多个特征与目标的相关性（控制年龄）
target = 'HM'
control_var = 'Age'

partial_results = []
for feature in df.columns:
    if feature not in [target, control_var]:
        corr, p_val = partial_spearman(df[feature], df[target], df[control_var])
        partial_results.append({
            'Feature': feature,
            'Partial_Spearman': corr,
            'P_Value': p_val,
            'Significance': '*' if p_val < 0.05 else ''
        })

partial_df = pd.DataFrame(partial_results)
partial_df = partial_df.sort_values(by='Partial_Spearman',
                                    key=lambda x: abs(x),
                                    ascending=False)

print("\n控制年龄后的部分Spearman相关结果:")
print(partial_df.head(10))


控制年龄后，BMI与HM的部分Spearman相关: -0.163 (p=0.1057)
控制年龄后的部分Spearman相关结果:
      Feature  Partial_Spearman       P_Value Significance
3  ODcataract         -0.860134  2.073595e-30            *
4  OScataract          0.737602  2.091370e-18            *
0         BMI         -0.162742  1.057138e-01
1         SBP          0.065917  5.146663e-01
2         DBP          0.012420  9.023913e-01
5      HEIGHT          0.008050  9.366455e-01
6      WEIGHT         -0.000138  9.989123e-01
```

### #4. 非参数相关矩阵的高级可视化

```
# 1. 创建相关矩阵聚类图
sns.clustermap(corr_matrix,
               cmap='coolwarm',
               center=0,
               annot=True,
               fmt=".2f",
               figsize=(14, 12),
               row_cluster=True,
               col_cluster=True)
plt.title('Clustered Spearman Correlation Matrix')
plt.tight_layout()
plt.savefig('clustered_correlation_matrix.png', dpi=300)
plt.show()

# 2. 创建相关网络图
import networkx as nx

plt.figure(figsize=(14, 12))
G = nx.Graph()

# 只添加显著相关的边 (|r| > 0.3 且 p < 0.05)
for i, col1 in enumerate(corr_matrix.columns):
    for j, col2 in enumerate(corr_matrix.columns):
        if i < j:  # 避免重复
            corr = corr_matrix.loc[col1, col2]
            p_val = p_matrix.loc[col1, col2]
            if abs(corr) > 0.3 and p_val < 0.05:
                G.add_edge(col1, col2, weight=abs(corr), correlation=corr)

# 节点大小基于连接数
node_size = [G.degree(node) * 100 for node in G.nodes()]

# 边宽基于相关强度
edge_width = [abs(data['correlation']) * 5 for _, _, data in G.edges(data=True)]

# 边颜色基于相关方向
edge_color = ['red' if data['correlation'] < 0 else 'blue' for _, _, data in G.edges(data=True)]

pos = nx.spring_layout(G, seed=42)
nx.draw_networkx_nodes(G, pos, node_size=node_size, node_color='lightblue', alpha=0.9)
nx.draw_networkx_edges(G, pos, width=edge_width, edge_color=edge_color, alpha=0.6)
nx.draw_networkx_labels(G, pos, font_size=10, font_weight='bold')

plt.title('Significant Spearman Correlations (|r| > 0.3, p < 0.05)')
plt.axis('off')
plt.tight_layout()
plt.savefig('correlation_network.png', dpi=300)
plt.show()
```

### #5. 结果解释与报告生成

```
def interpret_spearman(corr, p_val):
    """解释Spearman相关结果"""
    strength = ""
    direction = "positive" if corr > 0 else "negative"

    if abs(corr) >= 0.7:
        strength = "very strong"
    elif abs(corr) >= 0.5:
        strength = "strong"
    elif abs(corr) >= 0.3:
        strength = "moderate"
    elif abs(corr) >= 0.1:
        strength = "weak"
    else:
        strength = "no or negligible"

    significance = "statistically significant" if p_val < 0.05 else "not statistically significant"

    return f"{strength} {direction} correlation ({significance}, ρ={corr:.2f}, p={p_val:.4f})"

# 生成关键结果的解释报告
key_results = [
    ('BMI', 'HM'),
    ('SBP', 'DBP'),
    ('Age', 'ODcataract')
]

print("\n关键相关性结果解释:")
for var1, var2 in key_results:
    corr = corr_matrix.loc[var1, var2]
    p_val = p_matrix.loc[var1, var2]
    interpretation = interpret_spearman(corr, p_val)
    print(f"- {var1} 与 {var2} 之间: {interpretation}")

# 生成完整报告
with open('correlation_report.txt', 'w') as f:
    f.write("Spearman相关性分析报告\n")
    f.write("="*50 + "\n\n")

    f.write("1. 最强相关性:\n")
    top_corrs = corr_matrix.unstack().sort_values(key=abs, ascending=False)
    top_corrs = top_corrs[top_corrs.index.get_level_values(0) != top_corrs.index.get_level_values(1)]
    top_corrs = top_corrs.head(10)

    for idx, value in top_corrs.items():
        var1, var2 = idx
        p_val = p_matrix.loc[var1, var2]
        f.write(f"- {var1} 与 {var2}: ρ = {value:.3f}, p = {p_val:.5f}\n")

    f.write("\n2. 与目标变量(HM)最相关的特征:\n")
    hm_results = results.head(10)
    for _, row in hm_results.iterrows():
        f.write(f"- {row['Feature']}: ρ = {row['Spearman_Correlation']:.3f}, p = {row['P_Value']:.5f}\n")

    f.write("\n3. 方法说明:\n")
    f.write("使用Spearman秩相关系数(ρ)评估变量间的单调关系。\n")
    f.write("该非参数方法不假设线性关系或正态分布，适用于医学数据。\n")
    f.write("显著性水平设为α=0.05。")

print("\n分析报告已保存为 'correlation_report.txt'")


关键相关性结果解释:
- BMI 与 HM 之间: weak negative correlation (not statistically significant, ρ=-0.13, p=0.2038)
- SBP 与 DBP 之间: no or negligible negative correlation (not statistically significant, ρ=-0.05, p=0.6039)
- Age 与 ODcataract 之间: weak positive correlation (statistically significant, ρ=0.14, p=0.0000)

分析报告已保存为 'correlation_report.txt'


#退出虚拟环境study
conda deactivate


#删除虚拟环境
conda remove -n study --all
```
